# Human umbilical cord mesenchymal stem cell treatment alleviates symptoms in an atopic dermatitis-like mouse model

**DOI:** 10.1186/s13287-023-03365-w

**Published:** 2023-05-29

**Authors:** Chunting Hua, Qichang Liang, Siji Chen, Jiang Zhu, Yi Tang, Xianzhen Chen, Yinjing Song, Stijn van der Veen, Hao Cheng

**Affiliations:** 1 Department of Dermatology, Sir Run Run Shaw Hospital, School of Medicine, Zhejiang University, Hangzhou, China; 2 Department of Microbiology, Collaborative Innovation Center for Diagnosis and Treatment of Infectious Diseases, School of Medicine, Zhejiang University, Hangzhou, China; 3 Department of Dermatology, Zhejiang Provincial People’s Hospital, People’s Hospital of Hangzhou Medical College, Hangzhou, China

**Keywords:** Atopic dermatitis, Mesenchymal stem cells, Biomarkers

## Abstract

**Background:**

Atopic dermatitis (AD) is one of the most common immune and inflammatory skin disorders, leading to insufferable itching and skin abnormalities that seriously affect life quality of patients. There are still huge unmet needs for long-term and effective disease control, despite currently available therapies. Evidenced by some preclinical and clinical studies of AD treatment with stem cells, stem cell treatment could significantly and effectively ameliorate AD symptoms.

**Objectives:**

To elucidate underlying mechanisms of how stem cells therapy alleviates AD-like symptoms.

**Methods:**

An AD-like mouse model was constructed and treated with mesenchymal stem cells (MSCs) subcutaneously or subcutaneously combined with intravenously. The differentially expressed genes were sorted out from RNA sequencing results of dorsal skin and blood.

**Results:**

Two injection routes of MSCs could alleviate AD-like symptoms and pathologic changes of the skin and immune organs. RNA sequencing of dorsal skin sections and blood provided gene expression signatures for amelioration of skin defects, inflammatory and immune modulation by MSCs, as well as common AD molecular markers for the skin and blood, which may benefit for clinical diagnosis. IL-1β and its signaling pathway were specifically found to be associated with the development of AD-like dermatitis lesions. MSC treatment effectively inhibited the JAK-STAT pathway and receptors of IL-4, IL-13, IL-17, and IgE.

**Conclusions:**

MSC therapy could regulate abnormal immune and inflammatory status in AD. Mechanistic exploration will contribute to the development of personalized AD treatment based on MSCs.

**Supplementary Information:**

The online version contains supplementary material available at 10.1186/s13287-023-03365-w.

## Introduction

Atopic dermatitis (AD) is a common inflammatory skin disorder characterized by recurrent eczematous lesions and severe itching. Over the past 30 years, the prevalence of AD in children from developed countries has reached 10–20% [1], severely affecting life quality [2] and causing a high economic burden [3, 4, 5]. Many factors are associated with the development of AD, including genetic predisposition, epidermal barrier dysfunction, immune regulation disorders, abnormal skin microbiomes, and the neuroimmune system. Current theories suggest that very early and acute AD is almost exclusively characterized by Th2 cell infiltration, whilst a mixture of Th1, Th2, and Th22 cells are observed in chronic disease [6, 7, 8]. The skin of AD patients is susceptible to bacterial infections due to dysfunction of the epidermal barrier and dysregulation of skin antibacterial immunity [9]. Current treatments for AD include common systemic drugs like antihistamines, short-term oral glucocorticosteroids, and biological agents like dupilumab and nemolizumab [10, 11]. However, these traditional treatments are less effective and might have side effects in chronic and refractory AD [12]. Therefore, safer and more effective treatments need to be developed.

Mesenchymal stem cells (MSCs) are a group of cells that can be isolated from multiple tissues of mesodermal origin, and their vital properties, such as differentiation potential, ability to modulate immune responses, and low immunogenicity, make them ideal for the treatment of various diseases [13, 14, 15, 16]. One of the key benefits of MSCs is their intrinsic homing capabilities that stimulate migration to the injury site when administered systemically [17]. In a previous study, the addition of MSCs to differentiated effector T cells led to a decrease in the release of the pro-inflammatory cytokine interferon gamma (IFN-γ) from Th1 cells, with a concomitant increase in the release of interleukin-4 (IL-4) from Th2 cells, which has anti-inflammatory activities in Th1-mediated diseases [18]. Besides, in a murine model of ovalbumin-induced asthma, which is a Th2-mediated inflammatory disease [19], MSC-treatment attenuated airway hyper-responsiveness, reduced the number of eosinophils in bronchoalveolar lavage fluid and significantly decreased the release of Th2 cytokines. These findings suggest that MSCs regulate T cell immune responses, and cumulative data to date indicate that MSCs do not appear to inhibit immune cell functionality against infectious agents [20].

Cells derived from umbilical cord tissue are the youngest and most primitive MSCs available, with great potential to transform into any type of cell [21]. A promising clinical trial in South Korea, which involved subcutaneous implantation of umbilical cord MSCs for treatment of AD, has yielded promising results. Fifty-five percent of patients had a 50% reduction in the Eczema Area and Severity Index (EASI) and a 50% reduction in the Severity Score for Atopic Dermatitis (SCORAD). Those who received the higher dose also experienced a 50% reduction in itching [22].

In our current study, we established a 1-chloro-2,4-dinitrobenzene (DNCB)-induced AD-like mouse model and adopted human umbilical cord MSCs for exploration of curative effects. We found that MSC treatment can modulate inflammatory and immune response and improve skin barrier function, which breaks the vicious cycle of AD development. Common molecules in both skin and blood may further provide insights into AD diagnosis and treatment.

## Materials and methods

### Induction of AD-like lesions in BALB/c mice and MSC treatment

**Fig. 1:**
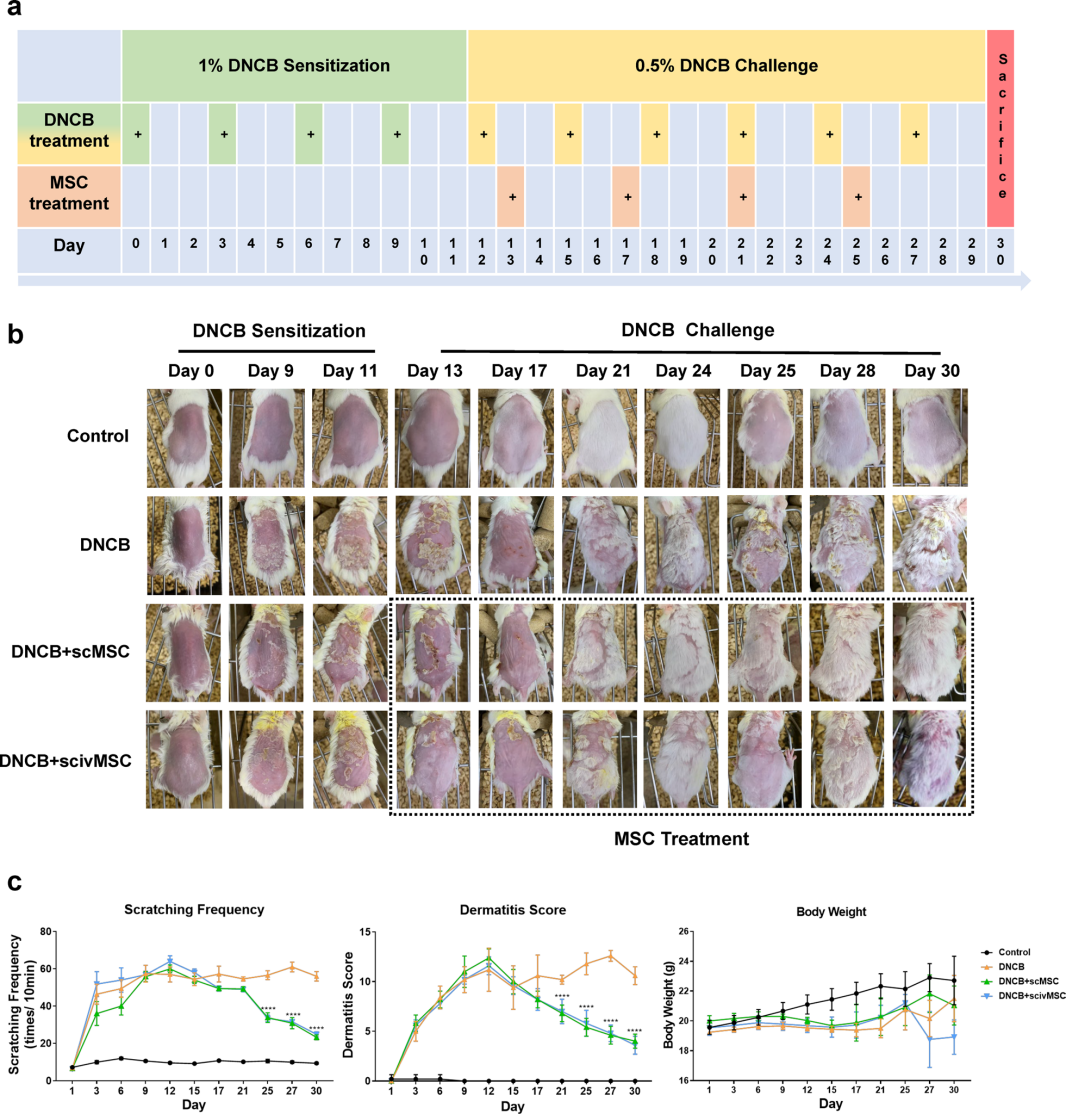
Effects of DNCB application and MSC treatment on the development of atopic dermatitis-like symptoms in BALB/c mice. **a** Schematic diagram of DNCB induction and MSC treatment from day 1 to day 30. 1% DNCB was applied on days 0, 3, 6, and 9, and 0.5% DNCB solution was applied on days 12, 15, 18, 21, 24, and 27. Mice received MSCs on days 13, 17, 21, and 25. **b** Macroscopic views of dorsal skin appearance from representative mice of each group from day 0 to day 30. **c** Records of scratching frequency, dermatitis score, and body weight of mice. Two-way ANOVA with Tukey’s multiple comparison test was used to test statistical significance between the DNCB group and MSC treatment groups. ****P < 0.0001

The procedures were performed according to the guidelines issued by Affairs Concerning Experimental Animals of the People’s Republic of China. Female six-week-old BALB/c mice were purchased from Shanghai SLAC Laboratory Animal Co., Ltd, and housed under specific pathogen-free conditions. DNCB and olive oil were obtained from Sigma. Twenty-four mice were divided randomly into four groups of six mice. The atopic dermatitis-like mouse model induced by DNCB, which activates the inflammasome, was established as previously described [23, 24, 25, 26], and adjusted according to our preliminary experiments. Briefly, the backs of mice were shaved with an electric clipper and treated with hair removal cream two days before sensitization. For sensitization, 1% DNCB was applied to the shaved area of the dorsal skin and the right ear on days 0, 3, 6, and 9. Mice of the solvent control group were treated with vehicle only (acetone: olive oil = 3:1 v/v). Subsequently, the dorsal skin and right ear were repeatedly challenged with 0.5% DNCB solution on days 12, 15, 18, 21, 24, and 27 (Fig. 1a). The optimal MSC dose was determined in our preliminary experiments (unpublished data). Each mouse in the MSC treatment group received a dose of 2 × 10^6^ MSCs in 100 μL culture solution on days 13, 17, 21, and 25. The cell suspensions were mixed gently before injection. For the scMSC group, 100 μL MSC solution was injected subcutaneously in the side of the dermatitis area. For the scivMSC group, 50 μL MSC solution was injected subcutaneously in the side of the dermatitis area and another 50 μL MSC solution was injected into the tail vein. The dorsal skin hair was shaved again on the day 24. All mice were euthanized by cervical dislocation on day 30.

### Dermatitis score and measurements

A comprehensive scoring system (Additional file 1: Table S1) adapted by Scoring Atopic Dermatitis (SCORAD) [27] was developed to measure the severity of skin symptoms by scoring erythema, edema, scales, lichenification, ear thickness and scratch frequency [28] with 0 (none), 1 (mild), 2 (moderate) and 3 (severe) [29, 30, 31, 32]. The scratch frequency was measured an hour after DNCB application with duration of ten minutes, and multiple scratches within one second were regarded as one event. Mouse body weight was measured every other day, and weight of the spleens and lymph nodes was measured after killing with electronic weighing scales.

### Histopathological analysis

Skin, spleen, and lymph node tissues were harvested on day 30 and fixed in formalin before embedding in paraffin. The procedures of deparaffinization, rehydration, antigen retrieval, and blocking were described previously [33]. Primary antibodies used for immunohistochemistry staining were Ki67 (#12202s, CST), CD4 (#25229S, CST), CD8 (#98941S, CST), CD1a (#17325-1-AP, Proteintech), CXCR3 (#26756-1-AP, Proteintech), CCR3 (#bs-1167R, Bioss). For toluidine staining, the hydrated samples were dyed with toluidine blue for 5 min, washed and differentiated with 1% hydrochloric acid alcohol. Images were obtained with a KFBIO Digital Slide Viewer or Olympus cellSens Entry 2.3. The mast cells were counted in five high-power fields (HPF) at X200.

### Measurement of serum IgE levels

Serum was separated from blood by centrifugation at 4000 rpm for 15 min and stored at − 80 °C until required. Serum IgE was tested with the IgE Mouse Uncoated ELISA Kit (# 88-50460, Invitrogen). Briefly, 96-well plates were coated with capture antibodies and blocked. Serial dilutions of standard IgE and diluted serum samples were added to the appropriate wells and incubated. The detection antibody, TMB, and stop solution were added before absorbance measurements at 450 and 570 nm.

### Immunofluorescence staining

Tissue sections were collected and embedded in OCT (#4583, SAKURA). Sections were washed with PBS and fixed with 4% paraformaldehyde (#P1110, Solarbio). Subsequently, they were permeabilized with buffered 1% (v/v) Triton X-100 (#T8200, Solarbio) and blocked with 10% goat serum. After blocking, the sections were labeled with antibodies against IL-1β (#AF5103, Affinity) using a 1:150 dilution and incubated overnight at 4 °C. Then, Alexa Fluor 594-conjugated secondary donkey anti-rabbit IgG (#34312, Yeasen) was used at a dilution of 1:300 and nuclei were stained with 4,6-diamidino-2-phenylindole (DAPI, #ab104139, Abcam). Immunofluorescence images were acquired using a fluorescence microscope (Olympus cellSens 2.3).

### RNA sequencing and statistical analysis

Dorsal skin sections and blood samples were collected on day 30, and total RNA was isolated with TRIzol reagent (#15596026, Invitrogen). RNA sequencing was conducted by LC-BIO Technologies Co., Ltd (Hangzhou, China). Gene Ontology (GO), Kyoto Encyclopedia of Genes and Genomes (KEGG) pathway enrichment were performed as previously described [34, 35]. The differentiated genes were selected by the criteria of |fold change|> 2 and *p* value < 0.5. Gene Set Enrichment Analysis was performed using the GSEA software (https://www.gsea-msigdb.org/gsea) [36, 37]. Gene expression was presented in a log_10_ scale. Fluorescence area was measured with ImageJ, and all results were analyzed using GraphPad Prism9. Statistical analysis includes one-way ANOVA or two-way ANOVA with Dunnett's multiple comparison test. Differences between the groups were considered significant at *P* < 0.05. *, *P* < 0.05; **, *P* < 0.01; ***, *P* < 0.001; ****, *P* < 0.0001.

## Results and discussion

### MSC treatment alleviates DNCB-induced atopic dermatitis-like symptoms in mice

**Fig. 2:**
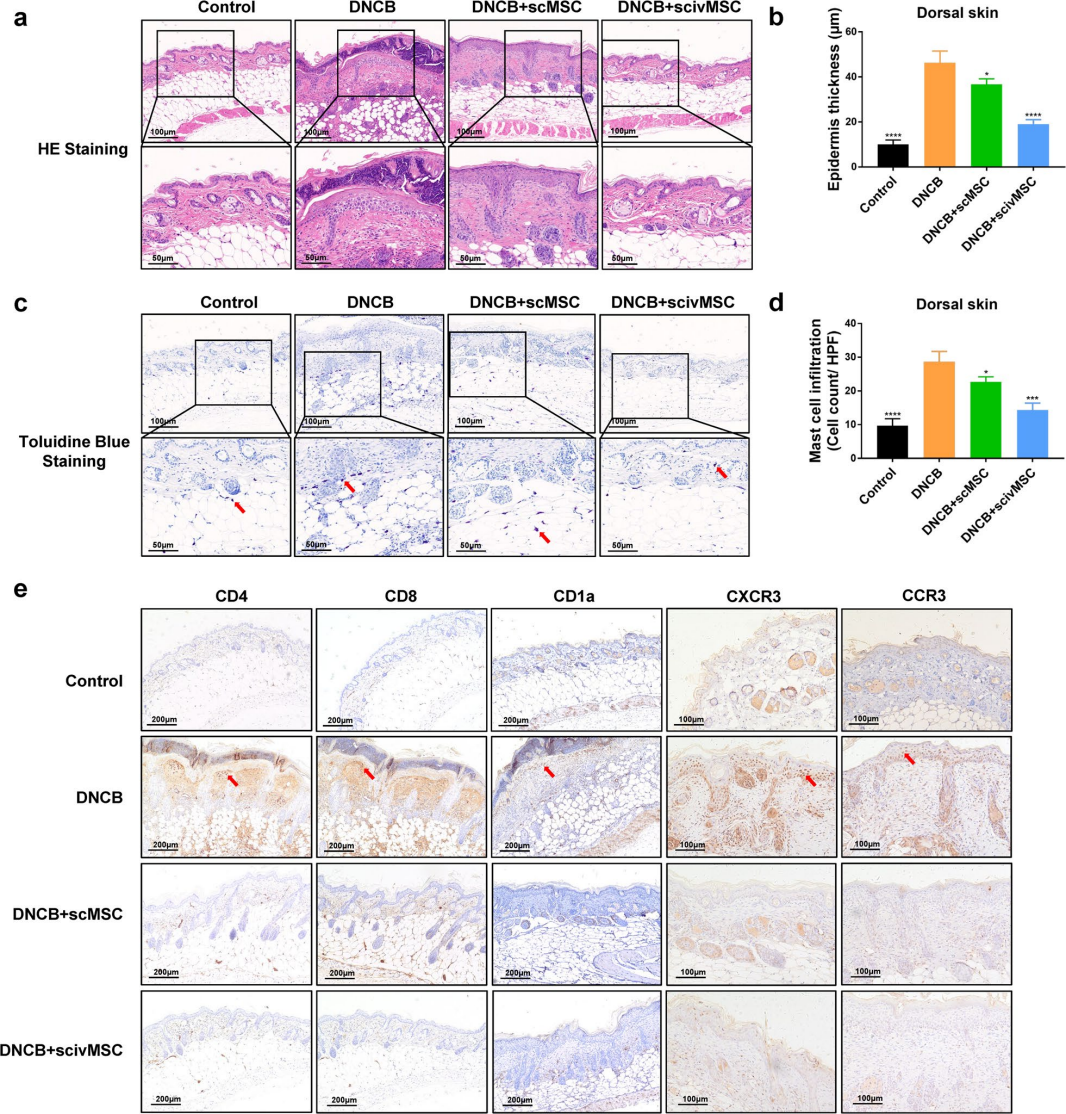
Pathological amelioration of epidermal changes by MSC treatment. **a** Visualization of histopathological manifestations in dorsal skin sections by hematoxylin–eosin staining. **b** Epidermis thickness based on tissue sections as presented in **a**. **c** Dorsal skin sections stained with toluidine blue to visualize mast cell infiltration. **d** Quantification of mast cell infiltration based on the number of dark violet-stained cells as presented in **c**. **e** Immunochemical staining of CD4, CD8, CD1a, CXCR3, and CCR3 of dorsal skin tissue sections. One-way ANOVA with Tukey’s multiple comparison test was used to test statistical significance of epidermal thickness and mast cell infiltration. *P < 0.05; ***P < 0.001; ****P < 0.0001

We evaluated the effects of MSC treatment in a mouse model of DNCB-induced AD. Briefly, mice were randomly divided into four groups, a control group, an AD-like disease group (DNCB group), and two MSC treatment groups receiving subcutaneous MSCs (DNCB + scMSC group) or subcutaneous and intravenous MSCs (DNCB + scivMSC group) one day following completion of the DNCB sensitization schedule (Fig. 1a). DNCB-induced symptoms were red, swollen skin with erythema, erosion, scaling, and dryness, resulting in elevation of the dermatitis score and increased scratching frequencies (Fig. 1b, c). MSC treatment initiated on day 13 gradually reduced symptoms, resulting in significantly reduced scratching frequencies and dermatitis scores by days 21–25. Similarly, mouse ear thickness, which reflects skin edema and epidermal hypertrophy, was increased after DNCB application and significantly reduced by MSC treatment from day 17 onwards (Additional file 2: Fig. S1a and b). The epidermis of both dorsal skin and the right ear was also markedly thickened after DNCB application, while epidermis thickening was significantly reduced after MSC treatment (Fig. 2a, b and Additional file 2: Fig. S1c and d). The skin sections of DNCB-challenged mice displayed hyperkeratosis with parakeratosis, acanthosis with mild spongiosis at the epidermis, vascular dilatation and hyperemia, and dermal infiltration of monocytes and multi-lobulated cells. MSC treatment ameliorated these symptoms, and inflammatory changes were mild or absent (Fig. 2a). Furthermore, toluidine staining is an established method for the identification and quantification of mast cells [38, 39], and the increased number and activation of mast cells are regarded as important AD pathogenesis factor [40, 41]. We found that DNCB-induced mast cell infiltration in the dorsal skin and right ear sections was significantly decreased after MSC treatment (Fig. 2c, d, Additional file 2: Fig. S1e and f).

Unexpectedly, hair coverage of the dorsal skin was higher in the MSC treatment groups compared with the DNCB group (Fig. 1b). Indeed, hair follicles containing Ki67-positive papilla cells were elevated after MSC treatment, which indicated that cell proliferation was promoted, and GSEA analysis performed for GO terms of dorsal skin sections also showed enrichment of hair follicle development after MSC treatment (Additional file 3: Fig. S2a and b).

### MSC treatment modulates inflammatory and immune response

Since AD is an inflammatory and immune skin disorder, mouse skin sections were analyzed by immunohistochemistry staining for immune cell markers. Compared with the control group, mice of the DNCB group showed a strikingly elevated infiltration by CD4^+^ and CD8^+^ T cells in the dermis (Fig. 2e), while this response was largely alleviated in the MSC treatment groups. Epidermal CD1a^+^ cells with a dendrite shape usually indicate Langerhans cells, which have shown to be critical for inflammatory skin diseases [42]. Although some researchers have suggested that CD1a is not expressed in mice [43], we found increased numbers of positive stained cells for CD1a in the hair follicles and dermis of mice from the DNCB group compared with control mice and mice from the MSC treatment groups (Fig. 2e). CXCR3 and CCR3 are regarded as selective Th1 and Th2 cell surface markers, respectively [44, 45]. A previous study found that the expression of CCR3 in the skin of AD patients was decreased after 8-week tacrolimus administration [46], which fits with the strong association between Th2 responses and acute AD. A DNCB challenge contributes to the chemotaxis of eosinophils expressing CCR3 and Th2 lymphocytes into the skin [47]; CXCR3 is upregulated during inflammation and recruitment of immune cells to inflammation sites [48]. Our results showed elevated levels of CXCR3^+^ and CCR3^+^ cells in the epidermis and dermis of the DNCB group, while immunoreactive cells for CXCR3 and CCR3 were reduced for the MSC treatment groups. Taken together, MSC treatment resulted in a major improvement of dermatitis-like skin lesions through immune and inflammatory modulation.

**Fig. 3:**
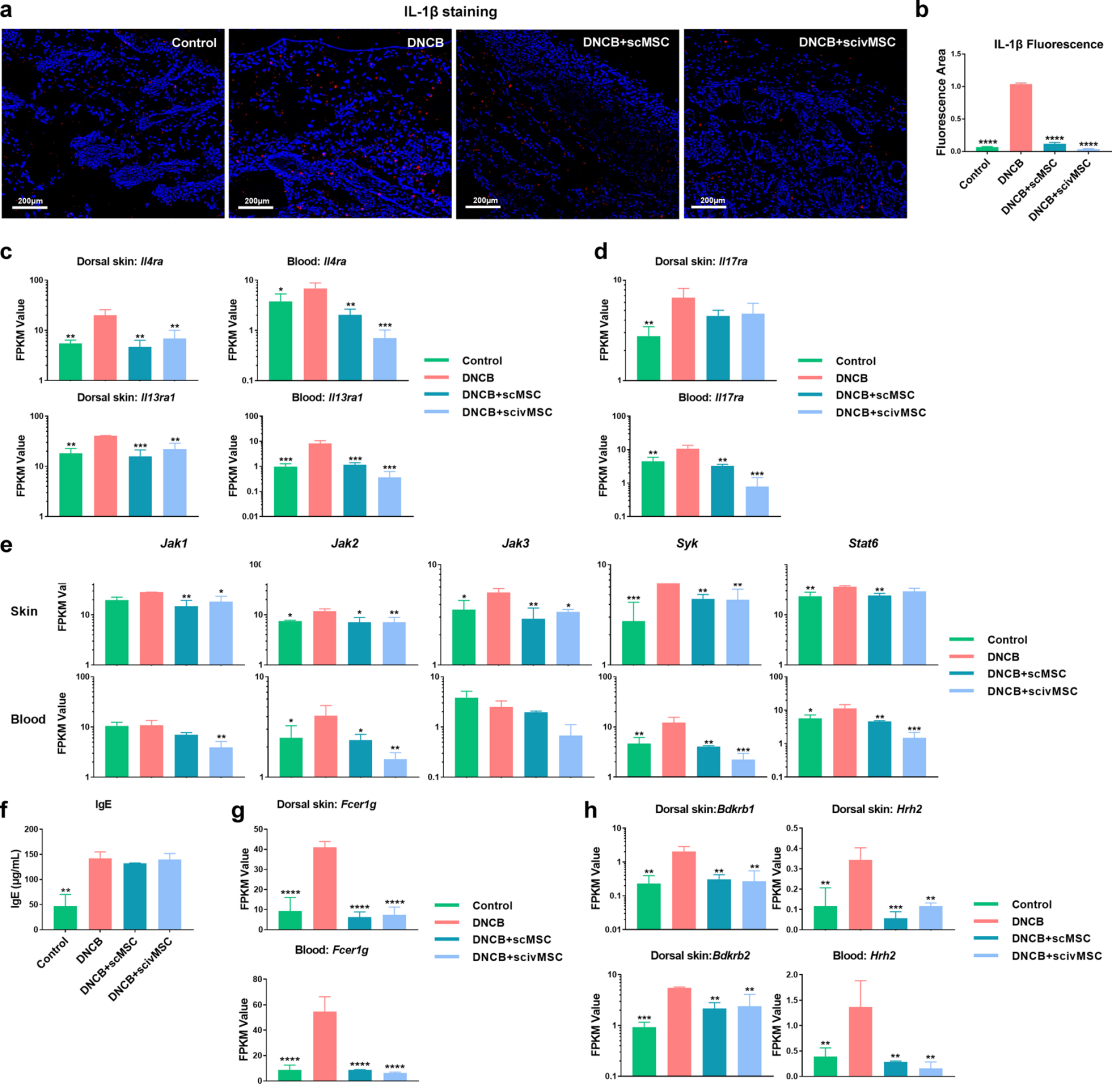
Modulation of inflammatory and immune response by MSC treatment. **a** Immunofluorescence staining of IL-1β (red) in dorsal skin tissue sections. **b** Quantification of IL-1β fluorescence area of tissue sections as presented in **a**. **c** Gene expression of *Il4ra* and *Il13ra1* in dorsal skin tissue sections and blood based on RNA seq. **d** Gene expression of *Il17ra* in dorsal skin tissue sections and blood based on RNA seq. **e** Gene expression of JAK-Stat pathway genes *Jak1*, *Jak2*, *Jak3*, *Syk* and *Stat6* in dorsal skin tissue sections and blood based on RNA seq. **f** Serum levels of IgE measured by ELISA. **g** Gene expression of the IgE receptor gamma chain *Fcer1g* in dorsal skin tissue sections and blood based on RNA seq. **h** Gene expression of *Bdkrb1, Bdkrb2* in dorsal skin, and *Hrh2* in dorsal skin tissue sections and blood based on RNA seq. Data are presented in a log10 scale. Significant differences compared with DNCB group were identified by one-way ANOVA with Tukey’s multiple comparison test. *P < 0.05; **P < 0.01; ***P < 0.001; ****P < 0.0001

RNA Seq-based transcriptomics of skin sections and blood and subsequent gene ontology (GO) enrichment analysis and gene set enrichment analysis (GSEA) performed for GO terms showed that genes related to inflammatory and immune responses were most enriched in the DNCB group compared with the MSC treatment groups (Additional file 4: Figure S3a, b and c), indicating that MSC treatment rebalances skin and blood homeostasis by modulation of inflammatory and immune responses. IL-1β is an important cytokine in AD development [49], and it is significantly elevated in stratum corneum tape strips [50] and interstitial fluid [51] from skin lesions of AD patients. Study concluded that IL-1 was released from epidermis with activation [52], and the increased level of skin IL1β was mainly derived from infiltrated neutrophils and monocytes/macrophages [53]. In our study, IL-1β gene expression was significantly higher in the DNCB group compared with the control and MSC treatment groups (Additional file 2: Figure S3d). Further immunofluorescence staining of dorsal skin sections also showed increased IL-1β levels for the DNCB group compared with the control and MSC treatment groups (Fig. 3a, b). IL-1RAP, IL-1R1 and IL-1R2 are IL1β receptors [54, 55], and IL-1β-IL-1R-MyD88 signaling has shown to be necessary for bacteria to promote skin regeneration [56]. What’s more, the NLRP3 also plays an important role in IL-1 related cytokine production [57, 58]. Consistently, gene expression levels of IL-1RAP, IL-1R1, IL-1R2, IL-1Rn (IL-1R antagonist), Myd88 and Nlrp3 were also specifically elevated in skin sections and/or blood for the DNCB group (Additional file 2: Figure S3d). Previous studies have shown that immune modulation by MSCs requires priming by inflammatory mediators [59, 60]; DNCB-mediated induction of IL-1β may therefore provide the trigger for MSCs to exert immunomodulatory effects.

In addition to traditional medicines, biological agents like dupilumab, which effectively antagonizes IL-4Rα and IL-13Rα, and JAK inhibitors like baricitinib, are currently used in moderate-to-severe AD patients. The JAK-STAT pathway is involved in the occurrence of AD skin inflammation, while inhibition will lead to a reduction in the release of pro-inflammatory cytokines such as IL-1 [61, 62]. Furthermore, a double-blind clinical trial recently validated the effectiveness of IL-17α antagonists secukinumab for treatment of AD patients [63]. In our study, although there was low expression level of IL-4 in both skin and blood and no detectable IL13 in skin, MSC treatment effectively reduced the gene expression levels of IL-4 and IL-13 receptors in both the skin and blood (Fig. 3c) and the IL-17 receptor in blood (Fig. 3d), which would likely exert similar effects as specific inhibitors. Similarly, we found that compared with the DNCB group, MSC treatment resulted in significantly reduced gene expression levels of *Jak1, Jak2* and *Jak3* in skin tissue sections and *Jak1* and *Jak2* in blood (Fig. 3e). Similar results were obtained for *Syk* and *Stat6*. Overall, compared with JAK inhibitors alone, MSCs may offer the advantage to modulate multiple cytokine signaling targets for treatment of AD.

IgE and its cascade are regarded as pivotal players in AD pathophysiology [64], and studies have shown some clinical benefit for use of the monoclonal anti-IgE antibody omalizumab in AD patients [65]. In our study, the level of serum IgE was increased in the DNCB induction groups, which was not alleviated by MSC treatment (Fig. 3f), while in a recent clinical trial MSC treatment was able to reduce serum IgE levels in AD patients [22]. However, in our study MSC treatment suppressed DNCB-induced expression of the IgE receptor *Fcer1g* in both skin sections and blood (Fig. 3g), which might alleviate IgE-based effects in AD.

Binding of histamine or bradykinin and their receptor can result in pain and pruritus, as well as cause mast cell degranulation and histamine release [66, 67]. As our results showed, the gene expression levels of histamine receptor H2 (*Hrh2*) in dorsal skin sections and blood, and bradykinin receptor 1 and 2 (*Bdkrb1* and *Bdkrb2*) in the dorsal skin were significantly increased by DNCB challenge and subsequently decreased after MSC treatment (Fig. 3h). Therefore, MSC treatment may interrupt the itch-scratch cycle by targeting neuroimmune receptors.

### MSC treatment alleviates DNCB-induced epidermal barrier dysfunction

**Fig. 4:**
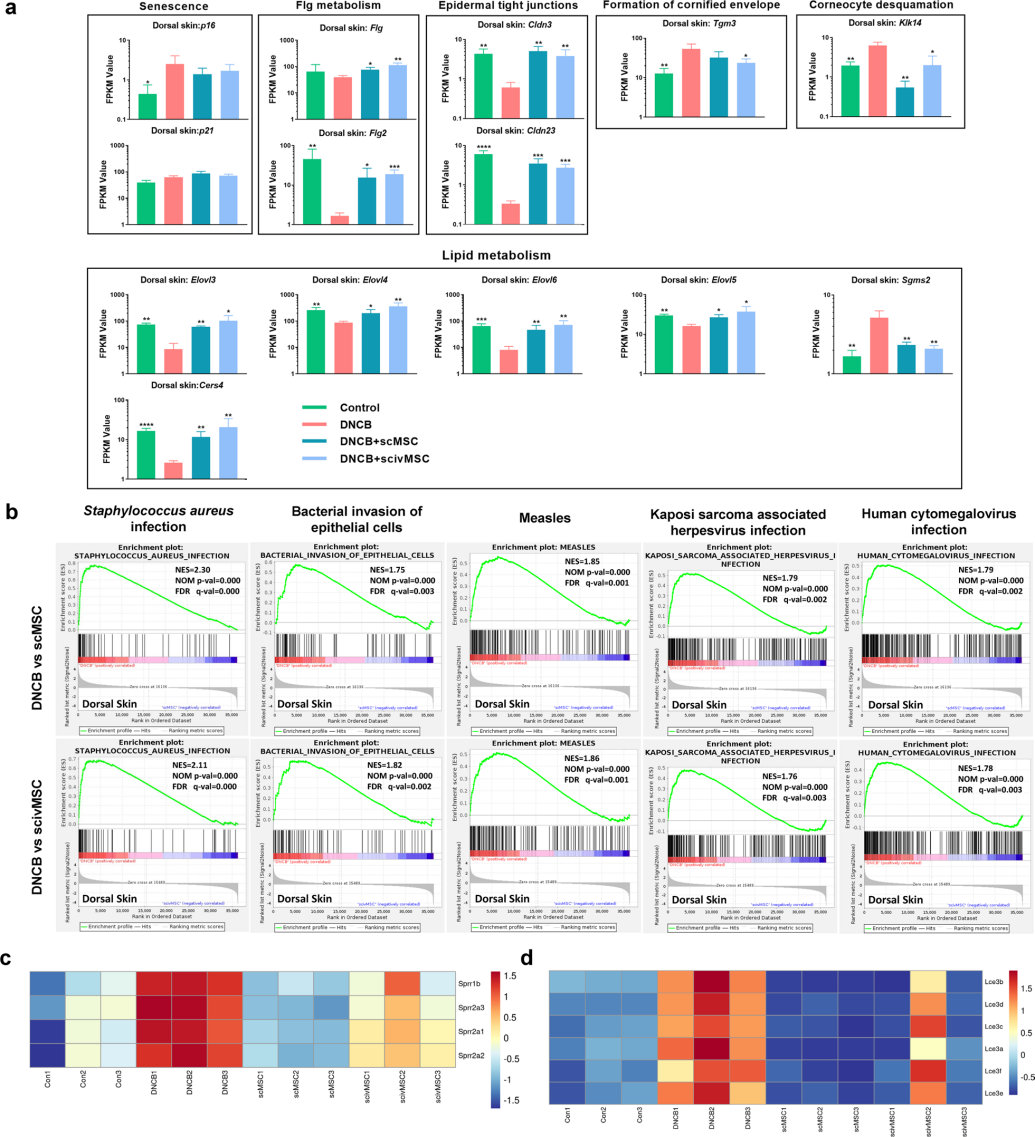
MSC treatment alleviates epidermal barrier dysfunction induced by DNCB application. **a** Gene expression of skin barrier function-related genes in dorsal skin tissue sections based on RNA seq. p16 and p21 are related to senescence; *Flg* and *Flg2* are related to filaggrin metabolism; *Cldn3* and *Cldn23* participate in epidermal tight junctions; *Tgm3* forms cornified envelope; *Klk14* contributes to corneocyte desquamation; *Elovl3*, *Elovl4*, *Elovl5*, *Elovl6*, *Sgms2* and Cers4 are involved in lipid metabolism. Significant differences compared with DNCB group were identified by one-way ANOVA with Tukey’s multiple comparison test. **b** GSEA analysis of dorsal skin tissue section RNA seq data performed for KEGG pathways *Staphylococcus aureus* infection, bacterial invasion of epithelial cells, Measles, Kaposi sarcoma-associated herpesvirus infection and human cytomegalovirus infection. **c** Heatmap of the transcriptome values for *Sprr1b*, *Sprr2a1*, *Sprr2a2* and *Sprr2a3*. **d** Heatmap of the transcriptome values for the *Lce3* family (genes a to f). *P < 0.05; **P < 0.01; ***P < 0.001

Most dermatitis is caused by senescence of the cells after either chemical or radiation exposure. For example, p16 and p21 are identified senescence markers [68, 69, 70]. The RNA Seq showed that DNCB application increased p16 level of dorsal skin, while p21 had no significant change (Fig. 4a). Besides, MSC treatment seemed to have limited effect on p16 and p21 in dorsal skin. Filaggrin is one of the most important epidermal proteins involved in the pathogenesis of AD, with specifically loss-of-function mutations being the most significant risk factor for AD [71]. In our study, *Flg* and *Flg2* gene expression was reduced in the DNCB group, while MSC treatment alleviated reduced expression (Fig. 4a). Consistent with DNBC-induced epidermal barrier dysfunction, *Cldn3* and *Cldn23*, involved in epidermal tight junction formation, and *Tgm3* and *Klk14*, involved in the formation of the cornified envelope and corneocyte desquamation, respectively, were all downregulated in the DNCB group, while MSC treatment restored expression patterns. Similarly, MSC treatment restored transcriptional expression of lipid metabolism-related genes such as *Elovl3, Elovl4, Elovl5, Elovl6, Sgms2 and Cers4* (Fig. 4a), which recovered intercellular lipid function and moisture retention ability by increasing ceramides content.

Skin infections are a major complication of AD and can be caused by impaired skin integrity. For example, *Staphylococcus aureus* colonizes on the skin of 60–100% of AD patients, compared with 5%–30% of healthy controls [72, 73]. Kaposi's varicelliform eruption [74] and active subclinical cytomegalovirus infection [75] occur with preexisting skin AD, which lead to aggravation or ineffective treatment. From the GSEA analysis performed for KEGG pathways of dorsal skin sections, pathways like *Staphylococcus aureus* infection, bacterial invasion of epithelial cells, measles, Kaposi sarcoma-associated herpesvirus infection and human cytomegalovirus infection were all specifically enriched in the DNCB group (NOM *P*-value < 0.05, FDR q-value < 0.25; Fig. 4b), further indicating that MSC treatment might counteract or prevent skin infections or reduce infection-induced inflammatory responses. Previous studies found that SPRR1B, SPPR2A and LCE3 were induced by bacterial exposure and might have antibacterial activity [76, 77]. As our RNA Seq showed, *Sprr1b, Sprr2 (a1 to a3)* and *Lce3*(a, b, c, d, e, f) were significantly upregulated in the DNCB group compared with the control and MSC treatment groups (Fig. 4c, d), which indicates that DNCB-induced skin barrier dysfunction might enhance bacterial colonization or infection of the skin, while MSC treatment stimulates repair of the skin barrier.

Further insights into the therapeutic potential of MSC may lead the development of innovative anti-infection treatments.

### Effects of MSC treatment on spleen and lymph node morphology and immune infiltration

**Fig. 5:**
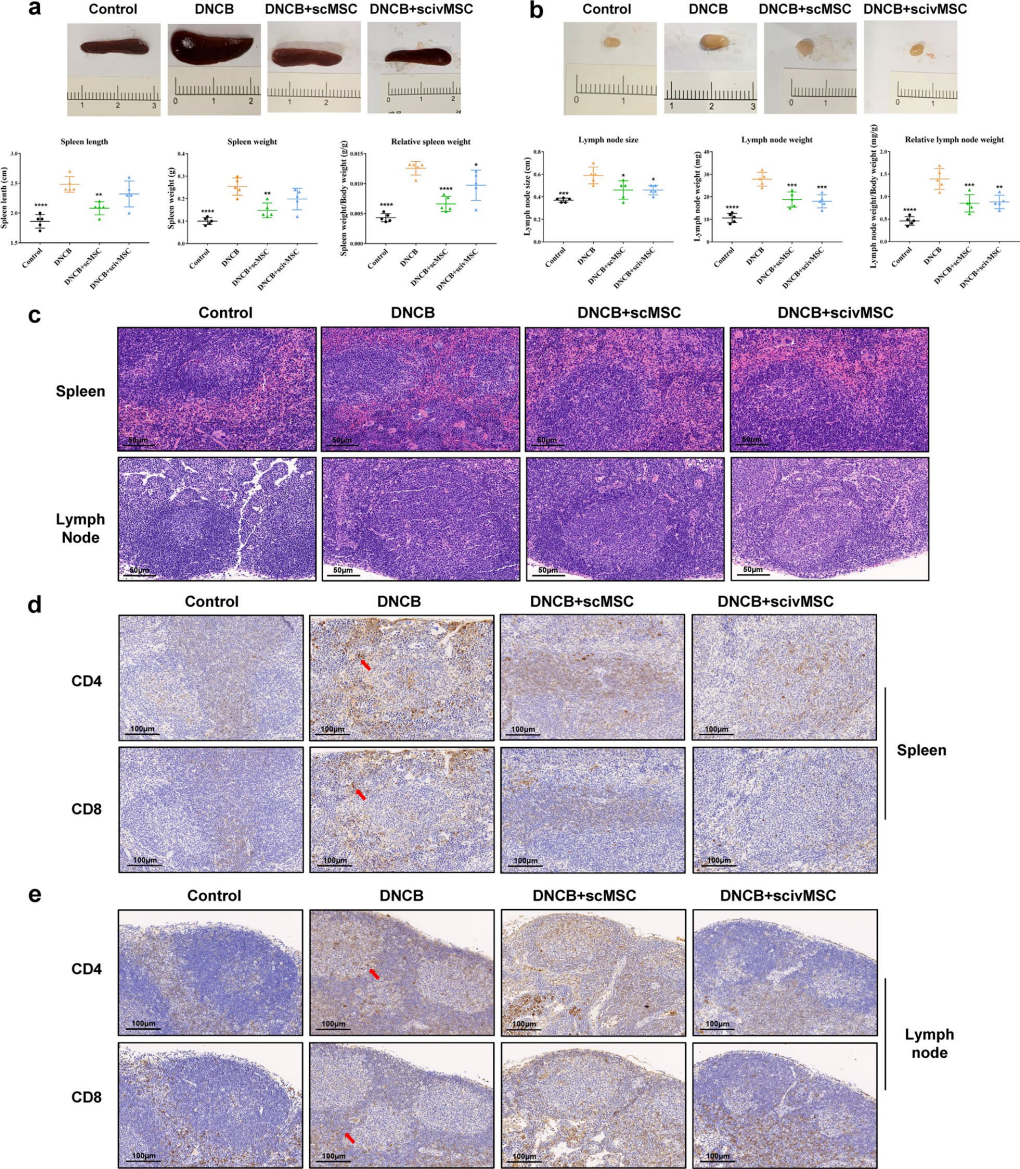
Morphology and pathology changes of spleen and lymph nodes after DNCB application and MSC treatment. **a** Representative images of the spleen for each group and quantification of spleen length, weight and relative weight. **b** Representative images of the axillary lymph node for each group and quantification of lymph node size, weight and relative weight. **c** HE staining of spleen and axillary lymph node sections. **d** CD4 and CD8 staining of spleen tissue sections. **e** CD4 and CD8 staining of lymph node tissue sections. One-way ANOVA with Tukey’s multiple comparison test was used to test statistical significance. *P < 0.05; **P < 0.01; ***P < 0.001; ****P < 0.0001

The spleen is the filter organ for blood and the largest lymphatic organ in the body, while lymph nodes collect antigenic substances. Our research showed that DNCB induced splenomegaly and enlarged lymph nodes, which indicated abnormality of the immune system (Fig. 5a, b), while the absolute or relative weight of the spleen and lymph nodes was significantly decreased after MSC treatment (Fig. 5a, b). Histopathology of the spleen showed basically a clear and organized structure in the control group, while for the DNCB group the spleen presented white pulp atrophy, hemorrhage and intermixed red and white pulp (Fig. 5c). These morphological changes were significantly reduced after MSC treatment, regardless of the MSC injection route. For the lymph nodes, primary lymphoid follicles can be observed in the control group, while secondary lymphatic follicles and germinal centers were seen in the DNCB group. Furthermore, the DNCB group showed lymphocytic hyperplasia of the lymph nodes and increased germinal centers. These morphological lymph node changes were alleviated after MSC treatment (Fig. 5c).

T lymphocytes are major players in the adaptive immune system, which proliferate and release inflammatory cytokines and chemokines. Previous studies found that CD4^+^ and CD8^+^ T lymphocyte subsets proliferated after DNCB treatment [78, 79], which is consistent with our findings. In both the spleen and lymph nodes, CD4^+^ and CD8^+^ T lymphocytes were increased in the white pulp or lymphatic follicles for the DNCB group, which was decreased after MSC treatment, indicating that MSC treatment can alleviate immune abnormalities (Fig. 5d, e).

### Common AD signatures in skin and blood

**Fig. 6:**
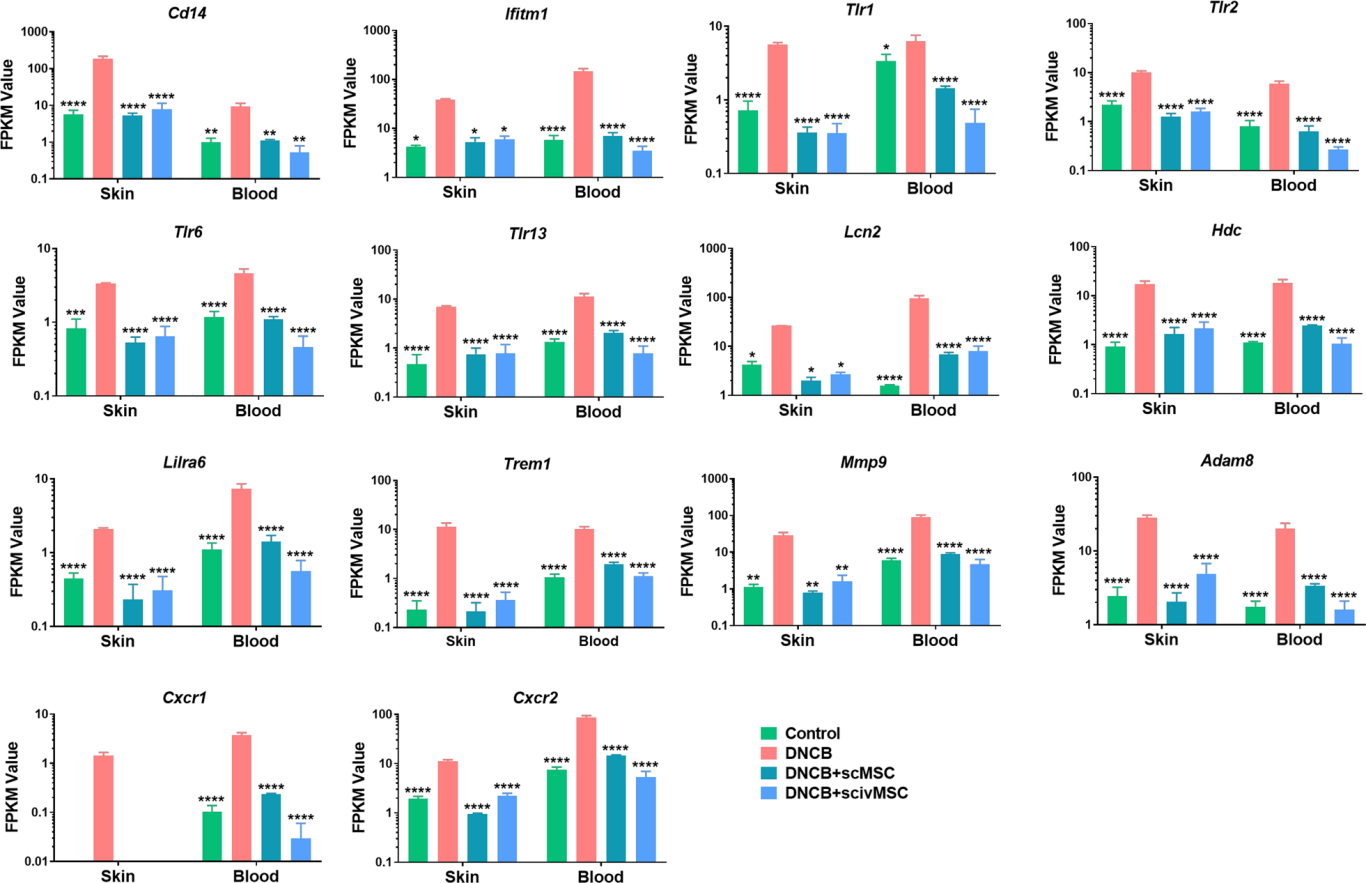
Common AD signatures in dorsal skin tissue sections and blood. Gene expression of *Cd14*, *Ifitm1*, *Tlr1*, *Trl2*, *Trl6*, *Tlr13*, *Lcn2*, *Hdc*, *Lilra6*, *Trem1*, *Mmp9*, *Adam8*, *Cxcr1* and *Cxcr2* in dorsal skin tissue sections and blood based on RNA seq. Apart from DNCB group, the expression level of Cxcr1 in skin was nearly undetectable. Data are presented in a log10 scale. Significant differences compared with DNCB group were identified by two-way ANOVA with Tukey’s multiple comparison test. *P < 0.05; **P < 0.01; ***P < 0.001; ****P < 0.0001

Since distinct clinical and molecular phenotypes may lead to different therapeutic outcomes, a shift to precision therapy may improve the management of AD. Due to the complexity of the disease, the potential of AD biomarkers has not been fully elucidated. A combination of clinical considerations and a reliable approach to the assessment of AD-specific biomarkers will further advance AD research and improve patient management [80]. Thus, specific molecules that correlate with disease severity in atopic dermatitis and show a similar pattern in both blood and the skin may be insightful for clinical diagnosis. A clinical trial (number NCT01782703) is currently recruiting and evaluates the importance of skin and blood biomarkers in pediatric AD. Skin and blood profiling may help formulate pathogenic hypotheses, find biomarkers for therapeutic responses, and monitor the effectiveness of MSC therapy in AD patients. We sorted out promising genes (Additional file 5: Fig. S4), which are listed with fold change in Additional file 1: Tables S2 and 3. CD14 [81, 82] and Ifitm1 [83] have previously been acclaimed as integral components of AD, and their high expression was induced in the DNCB group (Fig. 6), but restored to control levels after MSC treatment. Abnormal activation of TLRs promotes autoimmune and inflammatory disease development [84], while in our study, *Tlr1, Tlr2, Tlr6, Tlr13* were consistently overexpressed in the skin and blood after DNCB challenge, but rebalanced by MSC treatment (Fig. 6). DNA demethylation of Fcer1g [85] is related to overexpression of Fc receptor γ subunit (FcRγ)-related receptors on antigen-presenting cells. Lcn2 is an astrocytic STAT3-dependent inflammatory factor involved in maintenance of skin homeostasis and chronic itch [86, 87], and serum Lcn2 levels in AD patients are significantly higher compared with healthy subjects [88]. We found MSC treatment alleviates DNCB-induced *Fcer1g and Lcn2* expression (Figs. 3g, 6), which may help to repress allergen sensitization and allergic inflammation. Overexpression of Hdc [89] in keratinocytes that produce histamine was observed in AD patients, and Lilra6 overexpression [90] affects immune balance in AD patients. Furthermore, the Trem1 [91] pathway is activated in AD patients, and high Mmp9 [92] serum level is found in AD patients. In our results, the mRNA level of these AD-related risk factors was induced in the DNCB group and subsequently downregulated after MSC treatment. Another gene, Adam8, was found to be elevated in airway epithelium or airway inflammatory cells in mouse and human asthma patients [93], and dual CXCR1/CXCR2 [94] antagonist could alleviate airway neutrophilia in mild allergic asthma subjects, indicating their potential roles in allergic diseases regulation.

## Conclusions

AD is a chronic recurrent disease for which treatment is not curative, but instead alleviates symptoms by reducing inflammation, relieving itching, and improving the skin barrier. The DNCB-induced AD mouse model is symptom–based, with some differences with real AD pathogenesis. Epidermal barrier disorders, pathologic hyperinflammation, and immune infiltration were observed in DNCB-induced mice, which are common in AD and promote disease progression. These effects were reversed by both subcutaneous or/and intravenous treatment with MSCs, indicating that MSCs can be effective in ameliorating chronic skin inflammation and correcting the observed cellular derangement. We believe improvements of the regenerative capacity and immunomodulatory capacity provided by MSCs can significantly improve life quality of AD patients. Although there were many promising advanced therapies based on MSCs, more clinical studies are needed to assess safety and efficacy in the long-term effects. In future studies, their effects on pregnancy, curative effect prolongation, and personalized injection dose and mode should also be taken into consideration.

## Supplementary Information

**Additional file 1: Table S1**. Adjusted dermatitis score criteria. **Table S2**. Markers of negative correlation with AD severity. These genes are lowly expressed in DNCB group compared with MSC treatment groups. **Table S3**. Markers of positive correlation with AD severity. These genes are highly expressed in DNCB group compared with MSC treatment groups.

**Additional file 2: Fig. S1.** Morphology and pathological changes of right ear and its mast cell infiltration. **a** Macroscopic views of right ear appearance from representative mice in each group from day 0 to day 30. **b** Record of ear thickness during DNCB application and MSC treatment. **c** HE staining of right ear in different groups. **d** Measurements of epidermis thickness of right ear based on slides as presented in panel c. **e** Toluidine blue staining of right ear in different groups. **f** Mast cells infiltration of right ear based on slides as presented in panel. One-way ANOVA with Tukey’s multiple comparison test was used to test statistical significance. *, P < 0.05; **, P < 0.01; ***, P < 0.001; ****, P < 0.0001.

**Additional file 3: Fig. S2.** Effects on hair growth by MSC treatment in DNCB mice model. **a** Ki67 staining of dorsal skin sections. Positive staining indicates activated proliferation. **b** GSEA analysis related to hair follicle development of dorsal skin between DNCB and MSC treatment groups. One-way ANOVA with Tukey’s multiple comparison test was used to test statistical significance. *, P < 0.05; **, P < 0.01; ***, P < 0.001; ****, P < 0.0001.

**Additional file 4: Fig. S3.** Inflammatory and immune patterns changes based on RNA Seq. **a** GO enrichment analysis of dorsal skin between DNCB vs scMSC and DNCB vs scivMSC. **b** GO enrichment analysis of blood between DNCB vs scMSC and DNCB vs scivMSC. **c** GSEA analysis related to inflammatory, immune and innate immune response of dorsal skin between DNCB vs scMSC and DNCB vs scivMSC. **d** Transcriptome changes of IL1β, IL1RAP, IL1R1, IL1R2, IL1RN, Myd88 and Nlrp3 in dorsal skin and blood. One-way ANOVA with Tukey’s multiple comparison test was used to test statistical significance. *, P < 0.05; **, P < 0.01; ***, P < 0.001; ****, P < 0.0001.

**Additional file 5: Fig. S4.** Process of picking common AD signatures. **a** Secondary analysis of differentially expressed genes between DNCB vs scMSC and DNCB vs scivMSC in dorsal skin and blood. **b** Advanced volcano plots show gene correlation with different MSC treatment routes.Collection of genes with same variation tendency in different MSC treatment routes.

## Data Availability

The raw sequence datasets for this study can be found in the Gene Expression Omnibus. The datasets for dorsal skin and blood are GSE209704 and GSE209517, respectively.

## Abbreviations

AD: Atopic dermatitis
MCSs: Mesenchymal stem cells
IFN-γ: Interferon gamma
IL: Interleukin
EASI: Eczema Area and Severity Index
SCORAD: Severity Score for Atopic Dermatitis
DNCB: 1-Chloro-2,4-dinitrobenzene
sc: Subcutaneous
sciv: Subcutaneous and intravenous
GSEA: Gene set enrichment analysis
