# Supplementary material for: Human umbilical cord mesenchymal stem cell treatment alleviates symptoms in an atopic dermatitis-like mouse model
Source: Stem Cell Res Ther. 2023 May 29;14:147. doi: 10.1186/s13287-023-03365-w (PMC10227992; doi:10.1186/s13287-023-03365-w)
Supplement: Supplementary file 1 — Additional file 1: Table S1. Adjusted dermatitis score criteria. Table S2. Markers of negative correlation with AD severity. These genes are lowly expressed in DNCB group compared with MSC treatment groups. Table S3. Markers of positive correlation with AD severity. These genes are highly expressed in DNCB group compared with MSC treatment groups. [file 13287_2023_3365_MOESM1_ESM.docx]

**Supplementary Figure 1 | Morphology and pathological changes of right ear and its mast cell infiltration.** (a) Macroscopic views of right ear appearance from representative mice in each group from day 0 to day 30. (b) Record of ear thickness during DNCB application and MSC treatment. (c) HE staining of right ear in different groups. (d) Measurements of epidermis thickness of right ear based on slides as presented in panel (c). (e) Toluidine blue staining of right ear in different groups. (f) Mast cells infiltration of right ear based on slides as presented in panel (e). One-way ANOVA with Tukey’s multiple comparison test was used to test statistical significance. *, *P* < 0.05; **, *P* < 0.01; ***, *P* < 0.001; ****, *P* < 0.0001.

**Supplementary Figure 2 | Effects on hair growth by MSC treatment in DNCB mice model.** (a) Ki67 staining of dorsal skin sections. Positive staining (brown spots) indicates activated proliferation. (b) GSEA analysis related to hair follicle development of dorsal skin between DNCB and MSC treatment groups. One-way ANOVA with Tukey’s multiple comparison test was used to test statistical significance. *, *P* < 0.05; **, *P* < 0.01; ***, *P* < 0.001; ****, *P* < 0.0001.

**Supplementary Figure 3 | Inflammatory and immune patterns changes based on RNA Seq.** (a) GO enrichment analysis of dorsal skin between DNCB vs scMSC and DNCB vs scivMSC. (b) GO enrichment analysis of blood between DNCB vs scMSC and DNCB vs scivMSC. (c) GSEA analysis related to inflammatory, immune and innate immune response of dorsal skin between DNCB vs scMSC and DNCB vs scivMSC. (d) Transcriptome changes of *IL1β,* *IL1RAP, IL1R1, IL1R2, IL1RN, Myd88* and *Nlrp3* in dorsal skin and blood. One-way ANOVA with Tukey’s multiple comparison test was used to test statistical significance. *, *P* < 0.05; **, *P* < 0.01; ***, *P* < 0.001; ****, *P* < 0.0001.

**Supplementary Figure 4 |** **Process of picking common AD signatures.** (a) Secondary analysis of differentially expressed genes between DNCB vs scMSC and DNCB vs scivMSC in dorsal skin and blood. (b) Advanced volcano plots show gene correlation with different MSC treatment routes. (c) Collection of genes with same variation tendency in different MSC treatment routes.

**Supplementary Table 1 | Adjusted dermatitis score criteria.**

| Criterion/Score | 0 | 1 | 2 | 3 |
| --- | --- | --- | --- | --- |
| Erythema | None | Mild | Moderate | Severe |
| Edema |  |  |  |  |
| Scales |  |  |  |  |
| Lichenification |  |  |  |  |
| Ear thickness (centimeters) | ＜0.25 | 0.26-0.33 | 0.34-0.5 | ＞0.5 |
| Scratch Frequency (times/10 min) | ＜15 | 16-35 | 36-55 | ＞56 |

**Supplementary Table 2 | Markers of negative correlation with AD severity.** These genes are lowly expressed in DNCB group compared with MSC treatment groups.

| Gene | log2(FC) | | | |
| --- | --- | --- | --- | --- |
|  | Blood_sc | Blood_sciv | Skin_sc | Skin_sciv |
| Bcas3os1 | -2.08 | -2.01 | -9.18 | -14.82 |
| Bean1 | -0.84 | -1.35 | -1.42 | -1.05 |
| Bmp2 | -3.89 | -3.81 | -2.57 | -2.04 |
| Disp3 | -1.24 | -1.09 | -9.18 | -14.82 |
| Dlg3 | -1.98 | -1.18 | -0.66 | -0.67 |
| Dusp14 | -1.65 | -2.70 | -2.36 | -1.23 |
| Elovl6 | -1.28 | -2.86 | -2.51 | -3.15 |
| Fam57b | -1.64 | -1.55 | -1.79 | -1.97 |
| Gfi1b | -2.33 | -2.14 | -9.18 | -14.82 |
| Glrp1 | -2.33 | -1.97 | -9.18 | -14.82 |
| Gm13842 | -1.10 | -1.02 | -9.18 | -14.82 |
| Gm28650 | -2.30 | -1.76 | -9.18 | -14.82 |
| Gm37614 | -0.95 | -0.71 | -1.79 | -1.44 |
| Gm38414 | -1.56 | -1.07 | -9.18 | -14.82 |
| H2bc15 | -2.13 | -2.38 | -16.85 | -14.82 |
| Hsd17b14 | -1.30 | -1.36 | -4.24 | -2.91 |
| Lef1os1 | -1.74 | -0.65 | -3.44 | -2.06 |
| Pard3b | -1.58 | -1.57 | -1.17 | -0.89 |
| Pdk2 | -1.05 | -1.14 | -0.72 | -0.89 |
| Prg2 | -2.28 | -2.69 | -16.85 | -14.82 |
| Rasl10a | -1.15 | -0.81 | -1.62 | -1.87 |
| Spock2 | -2.23 | -0.85 | -0.73 | -1.33 |
| Tjp2 | -1.11 | -1.17 | -1.34 | -0.62 |
| Usp54 | -0.94 | -0.61 | -1.11 | -1.03 |
| Wnk4 | -0.94 | -1.03 | -1.29 | -1.16 |

**Supplementary Table 3 | Markers of positive correlation with AD severity.** These genes are highly expressed in DNCB group compared with MSC treatment groups.

| Gene | log2(FC) | | | |
| --- | --- | --- | --- | --- |
|  | Blood_sc | Blood_sciv | Skin_sc | Skin_sciv |
| 1600010M07Rik | 2.34 | 4.67 | 3.71 | 3.77 |
| 2610528A11Rik | 8.41 | 6.29 | 3.94 | 1.91 |
| 4631405J19Rik | 8.41 | 6.29 | 9.47 | 2.68 |
| 5430403N17Rik | 8.41 | 6.29 | 9.47 | 1.88 |
| 5830428M24Rik | 1.70 | 3.32 | 2.66 | 3.29 |
| 8030451A03Rik | 8.41 | 6.29 | 9.47 | 4.05 |
| 9830107B12Rik | 3.21 | 3.41 | 9.47 | 16.31 |
| A530064D06Rik | 3.64 | 4.95 | 9.47 | 16.31 |
| AA467197 | 4.00 | 3.29 | 2.21 | 2.88 |
| AB124611 | 2.69 | 3.99 | 3.01 | 2.61 |
| AC124502 | 8.41 | 6.29 | 9.47 | 16.31 |
| Acod1 | 4.36 | 6.29 | 7.67 | 5.50 |
| Actr2 | 1.26 | 2.30 | 0.65 | 0.63 |
| Adam12 | 8.41 | 6.29 | 3.14 | 2.45 |
| Adam19 | 2.48 | 4.00 | 1.23 | 1.09 |
| Adam8 | 2.56 | 3.65 | 3.77 | 2.51 |
| Adamts4 | 8.41 | 6.29 | 3.34 | 1.60 |
| Adamts5 | 8.41 | 6.29 | 1.17 | 1.11 |
| Adamts7 | 8.41 | 6.29 | 1.45 | 1.01 |
| Adgra2 | 8.41 | 6.29 | 1.80 | 2.32 |
| Adgre5 | 1.46 | 2.96 | 1.05 | 1.08 |
| Adgrg3 | 2.91 | 3.02 | 1.27 | 1.58 |
| Adra2a | 8.41 | 6.29 | 2.05 | 2.14 |
| AI467606 | 1.70 | 3.16 | 1.13 | 0.89 |
| AI839979 | 2.65 | 6.29 | 2.41 | 1.52 |
| Akna | 1.10 | 3.04 | 0.97 | 1.13 |
| Akr1b8 | 8.41 | 6.29 | 2.78 | 1.94 |
| Akr1c18 | 8.41 | 6.29 | 2.10 | 1.95 |
| Aldh1l2 | 8.41 | 6.29 | 3.17 | 2.11 |
| Aldh3b1 | 2.96 | 3.57 | 1.60 | 1.82 |
| Alox5ap | 2.33 | 2.60 | 1.94 | 1.64 |
| Amdhd2 | 2.22 | 3.39 | 0.98 | 0.82 |
| Angpt2 | 8.41 | 6.29 | 1.33 | 1.19 |
| Angpt4 | 8.41 | 6.29 | 2.42 | 2.34 |
| Angptl1 | 8.41 | 6.29 | 1.21 | 1.58 |
| Ankk1 | 8.41 | 6.29 | 2.17 | 1.23 |
| Ankrd1 | 8.41 | 6.29 | 1.84 | 2.10 |
| Antxr2 | 2.12 | 3.78 | 1.45 | 1.20 |
| Anxa3 | 3.38 | 3.06 | 0.94 | 1.05 |
| Apba1 | 8.41 | 6.29 | 1.05 | 1.08 |
| Apbb1ip | 1.47 | 3.07 | 1.14 | 1.03 |
| Apln | 8.41 | 6.29 | 3.46 | 2.45 |
| Aplnr | 8.41 | 6.29 | 2.68 | 3.00 |
| Apobec1 | 2.51 | 3.87 | 1.20 | 1.12 |
| Apobr | 2.28 | 3.86 | 1.87 | 1.50 |
| Arap3 | 1.69 | 2.50 | 1.07 | 0.74 |
| Arg1 | 8.41 | 6.29 | 1.15 | 1.78 |
| Arg2 | 2.36 | 4.30 | 1.63 | 1.16 |
| Arhgap25 | 1.83 | 3.85 | 1.14 | 1.18 |
| Arhgap30 | 1.17 | 2.87 | 1.19 | 0.97 |
| Arhgap4 | 0.95 | 2.36 | 1.32 | 1.11 |
| Arhgap9 | 1.71 | 3.00 | 1.15 | 1.18 |
| Arid5a | 1.70 | 4.12 | 1.29 | 0.87 |
| Arl11 | 3.27 | 4.47 | 2.56 | 2.89 |
| Armh2 | 8.41 | 6.29 | 9.47 | 16.31 |
| Arpc1b | 1.78 | 2.65 | 1.17 | 0.86 |
| Arpc4 | 1.50 | 2.53 | 0.60 | 0.75 |
| Arsi | 8.41 | 6.29 | 1.61 | 1.87 |
| Aspn | 8.41 | 6.29 | 1.43 | 1.37 |
| Atp8b4 | 2.12 | 4.24 | 2.21 | 1.57 |
| B230303A05Rik | 8.41 | 6.29 | 9.47 | 16.31 |
| B3gnt9 | 8.41 | 6.29 | 1.84 | 2.25 |
| B430306N03Rik | 2.33 | 3.64 | 3.53 | 3.10 |
| B4galt2 | 8.41 | 6.29 | 1.14 | 1.19 |
| B4galt5 | 1.33 | 3.53 | 1.33 | 1.32 |
| Baalc | 8.41 | 6.29 | 2.61 | 1.35 |
| BC106175 | 8.41 | 6.29 | 3.62 | 16.31 |
| Bcl2l15 | 8.41 | 6.29 | 2.38 | 1.70 |
| Bcl3 | 2.21 | 2.98 | 1.95 | 1.36 |
| Bcl6b | 8.41 | 6.29 | 1.23 | 1.45 |
| Bdkrb1 | 8.41 | 6.29 | 2.70 | 2.91 |
| Birc3 | 1.60 | 3.11 | 0.98 | 0.70 |
| Bst1 | 2.91 | 4.48 | 2.64 | 2.19 |
| C130026I21Rik | 3.17 | 3.58 | 2.62 | 2.11 |
| C1qtnf2 | 8.41 | 6.29 | 1.92 | 2.21 |
| C1qtnf3 | 8.41 | 6.29 | 1.15 | 1.47 |
| C1qtnf5 | 8.41 | 6.29 | 2.14 | 2.23 |
| C1rl | 8.41 | 6.29 | 0.97 | 1.18 |
| C5ar1 | 2.90 | 4.05 | 2.05 | 1.66 |
| Cacna1c | 8.41 | 6.29 | 1.28 | 1.12 |
| Camp | 3.14 | 2.58 | 9.47 | 16.31 |
| Capn6 | 8.41 | 6.29 | 1.43 | 1.48 |
| Car9 | 8.41 | 6.29 | 2.15 | 1.78 |
| Cask | 2.39 | 2.69 | 0.80 | 0.70 |
| Casp4 | 2.58 | 3.85 | 1.27 | 0.90 |
| Catip | 8.41 | 6.29 | 1.57 | 1.79 |
| Ccbe1 | 8.41 | 6.29 | 2.62 | 2.33 |
| Ccdc102a | 8.41 | 6.29 | 1.03 | 1.26 |
| Ccdc88b | 1.55 | 2.85 | 2.09 | 1.71 |
| Cckar | 8.41 | 6.29 | 9.47 | 16.31 |
| Ccl19 | 8.41 | 6.29 | 9.47 | 16.31 |
| Ccn1 | 8.41 | 6.29 | 2.06 | 1.49 |
| Ccn4 | 8.41 | 6.29 | 3.49 | 3.12 |
| Ccno | 3.86 | 3.55 | 9.47 | 16.31 |
| Ccr1 | 2.25 | 3.51 | 4.60 | 3.97 |
| Ccr2 | 1.09 | 2.52 | 1.63 | 1.59 |
| Cd101 | 1.76 | 3.73 | 4.30 | 2.40 |
| Cd14 | 3.09 | 4.16 | 5.13 | 4.55 |
| Cd177 | 4.10 | 3.89 | 9.47 | 16.31 |
| Cd200r1 | 2.24 | 3.59 | 1.55 | 1.63 |
| Cd248 | 8.41 | 6.29 | 1.53 | 1.71 |
| Cd276 | 8.41 | 6.29 | 1.67 | 1.34 |
| Cd300a | 1.21 | 3.55 | 1.57 | 1.45 |
| Cd300c2 | 1.92 | 6.29 | 2.80 | 2.70 |
| Cd300lb | 2.43 | 3.84 | 3.00 | 16.31 |
| Cd300ld | 2.92 | 4.76 | 9.47 | 16.31 |
| Cd300ld5 | 8.41 | 6.29 | 1.70 | 1.59 |
| Cd300lf | 2.63 | 4.43 | 6.19 | 5.43 |
| Cd33 | 2.36 | 3.94 | 2.51 | 2.11 |
| Cd37 | 1.70 | 2.72 | 1.56 | 1.22 |
| Cd44 | 1.20 | 2.57 | 1.48 | 1.62 |
| Cd52 | 1.91 | 3.20 | 1.41 | 1.12 |
| Cd53 | 1.46 | 2.89 | 3.50 | 3.25 |
| Cd5l | 8.41 | 6.29 | 2.87 | 2.50 |
| Cd68 | 2.46 | 3.97 | 1.69 | 1.53 |
| Cdh11 | 8.41 | 6.29 | 1.82 | 1.77 |
| Cdh5 | 8.41 | 6.29 | 1.18 | 1.16 |
| Cdhr1 | 8.41 | 6.29 | 4.94 | 2.60 |
| Ceacam10 | 3.97 | 3.51 | 9.47 | 16.31 |
| Cfb | 8.41 | 6.29 | 1.33 | 1.28 |
| Cfp | 3.08 | 3.41 | 1.51 | 1.16 |
| Chil1 | 3.96 | 4.77 | 1.03 | 0.78 |
| Chil5 | 3.12 | 6.29 | 9.47 | 16.31 |
| Chst2 | 8.41 | 6.29 | 1.98 | 1.39 |
| Chst7 | 8.41 | 6.29 | 1.50 | 1.85 |
| Chsy1 | 1.79 | 3.32 | 1.02 | 1.07 |
| Ckap4 | 3.20 | 3.79 | 1.24 | 1.08 |
| Clec12a | 3.15 | 3.99 | 2.68 | 2.76 |
| Clec14a | 8.41 | 6.29 | 1.19 | 1.33 |
| Clec2e | 8.41 | 6.29 | 3.20 | 2.08 |
| Clec4a1 | 1.93 | 6.29 | 1.03 | 1.21 |
| Clec4a2 | 3.23 | 4.38 | 1.83 | 2.17 |
| Clec4a3 | 1.89 | 4.46 | 1.72 | 1.65 |
| Clec4d | 2.20 | 2.96 | 5.67 | 4.36 |
| Clec4e | 2.63 | 3.88 | 5.13 | 4.13 |
| Clec5a | 2.68 | 3.68 | 3.54 | 3.15 |
| Clec7a | 2.64 | 4.32 | 3.82 | 3.00 |
| Clic1 | 1.80 | 2.47 | 0.87 | 0.59 |
| Cln3 | 1.91 | 3.08 | 0.62 | 0.62 |
| Cmtm7 | 2.05 | 3.35 | 1.43 | 1.23 |
| Col12a1 | 8.41 | 6.29 | 3.53 | 3.37 |
| Col24a1 | 8.41 | 6.29 | 2.09 | 1.87 |
| Col5a1 | 2.01 | 6.29 | 2.50 | 2.04 |
| Col5a3 | 8.41 | 6.29 | 1.59 | 1.40 |
| Col8a1 | 8.41 | 6.29 | 2.46 | 2.42 |
| Coro1a | 1.59 | 2.78 | 2.31 | 1.80 |
| Cotl1 | 2.04 | 2.97 | 2.51 | 1.99 |
| Cpne2 | 3.47 | 3.97 | 1.73 | 1.21 |
| Cpz | 8.41 | 6.29 | 2.87 | 1.99 |
| Crabp1 | 8.41 | 6.29 | 2.96 | 2.76 |
| Creb3l3 | 8.41 | 6.29 | 2.54 | 2.27 |
| Crispld2 | 2.28 | 3.33 | 1.68 | 1.50 |
| Csf1 | 1.08 | 2.66 | 1.31 | 1.18 |
| Csf1r | 2.21 | 4.23 | 1.03 | 1.13 |
| Csf1r-ps | 2.49 | 2.93 | 9.47 | 16.31 |
| Csf2rb | 1.59 | 3.91 | 1.64 | 1.44 |
| Csf2rb2 | 2.82 | 3.93 | 1.74 | 1.28 |
| Csf3r | 2.44 | 4.23 | 4.84 | 4.29 |
| Csta1 | 8.41 | 6.29 | 1.76 | 1.00 |
| Cstdc4 | 4.57 | 5.35 | 6.04 | 4.85 |
| Cstdc5 | 4.96 | 5.04 | 3.85 | 1.69 |
| Cstdc6 | 8.41 | 6.29 | 3.95 | 1.62 |
| Ctsc | 1.75 | 2.52 | 0.78 | 1.04 |
| Ctss | 1.48 | 3.51 | 2.25 | 2.02 |
| Cxcl2 | 8.41 | 6.29 | 6.52 | 4.88 |
| Cxcr1 | 4.00 | 6.29 | 9.47 | 16.31 |
| Cxcr2 | 2.55 | 4.02 | 3.56 | 2.34 |
| Cxcr4 | 0.95 | 2.24 | 1.61 | 1.43 |
| Cyba | 2.11 | 2.84 | 1.69 | 1.61 |
| Cyp2u1 | 8.41 | 6.29 | 1.23 | 1.56 |
| Cyp4f18 | 2.26 | 3.43 | 5.42 | 5.42 |
| Cyp7b1 | 8.41 | 6.29 | 2.43 | 2.15 |
| Cyth4 | 1.61 | 3.29 | 2.12 | 2.17 |
| Cytip | 1.07 | 2.69 | 1.43 | 0.90 |
| Dchs1 | 8.41 | 6.29 | 1.47 | 1.74 |
| Dclk1 | 8.41 | 6.29 | 1.78 | 1.72 |
| Ddah1 | 8.41 | 6.29 | 1.99 | 1.92 |
| Ddias | 8.41 | 6.29 | 1.13 | 1.02 |
| Degs2 | 8.41 | 6.29 | 3.31 | 1.66 |
| Dennd1c | 1.11 | 2.67 | 1.51 | 1.02 |
| Dennd3 | 1.56 | 3.14 | 1.13 | 1.25 |
| Dhx58 | 2.25 | 4.70 | 1.70 | 1.21 |
| Dok3 | 2.49 | 3.60 | 1.61 | 1.05 |
| Dram1 | 2.18 | 6.29 | 1.17 | 1.11 |
| Dsel | 8.41 | 6.29 | 1.00 | 1.25 |
| Dusp9 | 8.41 | 6.29 | 1.50 | 1.92 |
| Dynap | 8.41 | 6.29 | 9.47 | 16.31 |
| Ecrg4 | 8.41 | 6.29 | 9.47 | 16.31 |
| Ecscr | 8.41 | 6.29 | 1.70 | 1.27 |
| Efhd2 | 1.89 | 3.55 | 1.23 | 1.11 |
| Egfl7 | 8.41 | 6.29 | 1.28 | 1.07 |
| Emilin2 | 2.20 | 3.84 | 1.55 | 1.39 |
| Eno1 | 1.71 | 2.84 | 1.76 | 1.25 |
| Eno1b | 1.77 | 3.58 | 1.95 | 1.97 |
| Entpd1 | 1.89 | 3.66 | 1.90 | 1.60 |
| Erich3 | 8.41 | 6.29 | 3.27 | 2.07 |
| F630028O10Rik | 2.30 | 3.74 | 4.59 | 3.52 |
| F830016B08Rik | 8.41 | 6.29 | 2.01 | 3.19 |
| Fabp5 | 8.41 | 6.29 | 2.64 | 1.48 |
| Fam114a1 | 2.95 | 3.32 | 1.43 | 1.44 |
| Fam167b | 8.41 | 6.29 | 2.14 | 1.93 |
| Fam20c | 8.41 | 6.29 | 0.97 | 1.16 |
| Fam49b | 1.66 | 2.81 | 0.99 | 0.92 |
| Fam71f2 | 8.41 | 6.29 | 4.43 | 2.26 |
| Fas | 1.70 | 2.96 | 1.04 | 0.79 |
| Fbln2 | 8.41 | 6.29 | 2.11 | 1.82 |
| Fbxl7 | 8.41 | 6.29 | 1.37 | 1.18 |
| Fcer1g | 2.66 | 3.15 | 2.71 | 2.49 |
| Fcgr1 | 1.77 | 6.29 | 2.36 | 2.24 |
| Fcgr2b | 1.71 | 3.17 | 1.87 | 1.59 |
| Fcgr3 | 2.18 | 3.48 | 2.03 | 2.01 |
| Fcgr4 | 2.40 | 3.87 | 3.71 | 2.81 |
| Fcrls | 8.41 | 6.29 | 1.16 | 2.07 |
| Fes | 2.28 | 3.70 | 1.43 | 1.22 |
| Fetub | 8.41 | 6.29 | 2.10 | 1.52 |
| Fgd3 | 1.43 | 2.71 | 2.24 | 2.31 |
| Fgr | 2.68 | 4.25 | 3.86 | 2.98 |
| Fibin | 8.41 | 6.29 | 2.04 | 1.79 |
| Fkbp11 | 8.41 | 6.29 | 1.36 | 1.18 |
| Fkbp14 | 8.41 | 6.29 | 1.51 | 1.38 |
| Fkbp7 | 8.41 | 6.29 | 1.44 | 1.21 |
| Flt4 | 8.41 | 6.29 | 1.45 | 1.27 |
| Fmnl1 | 1.52 | 2.97 | 1.99 | 1.89 |
| Fndc3b | 1.71 | 4.52 | 1.29 | 1.04 |
| Fosl1 | 8.41 | 6.29 | 3.09 | 2.28 |
| Fpr1 | 3.72 | 5.13 | 9.47 | 6.54 |
| Fpr2 | 3.73 | 5.12 | 9.47 | 5.83 |
| Fxyd5 | 1.47 | 2.73 | 1.92 | 1.49 |
| G0s2 | 2.84 | 6.29 | 3.35 | 3.18 |
| Gad1-ps | 1.60 | 3.29 | 3.04 | 16.31 |
| Gadd45b | 1.63 | 2.99 | 1.10 | 1.40 |
| Galns | 2.47 | 3.78 | 1.68 | 1.98 |
| Gas1 | 8.41 | 6.29 | 1.19 | 1.08 |
| Gdnf | 8.41 | 6.29 | 1.10 | 1.41 |
| Gfra1 | 8.41 | 6.29 | 1.66 | 2.13 |
| Gja5 | 3.25 | 6.29 | 0.83 | 0.93 |
| Gjb6 | 8.41 | 6.29 | 1.17 | 1.34 |
| Gk | 3.14 | 3.19 | 1.19 | 1.13 |
| Gla | 2.32 | 6.29 | 0.96 | 1.40 |
| Glipr1 | 1.91 | 2.75 | 3.28 | 2.40 |
| Glipr2 | 2.05 | 2.77 | 1.96 | 1.72 |
| Gm10108 | 8.41 | 6.29 | 3.07 | 2.90 |
| Gm10143 | 8.41 | 6.29 | 2.59 | 2.86 |
| Gm10913 | 8.41 | 6.29 | 9.47 | 16.31 |
| Gm11714 | 8.41 | 6.29 | 9.47 | 4.79 |
| Gm13748 | 8.41 | 6.29 | 9.47 | 16.31 |
| Gm14328 | 8.41 | 6.29 | 2.50 | 1.50 |
| Gm14548 | 2.91 | 4.25 | 9.47 | 16.31 |
| Gm15448 | 2.42 | 6.29 | 9.47 | 16.31 |
| Gm15832 | 1.73 | 3.55 | 1.36 | 1.75 |
| Gm15845 | 8.41 | 6.29 | 2.46 | 2.20 |
| Gm15922 | 2.87 | 4.03 | 3.66 | 3.35 |
| Gm15931 | 2.33 | 3.79 | 2.38 | 3.08 |
| Gm16026 | 3.22 | 6.29 | 9.47 | 16.31 |
| Gm18787 | 8.41 | 6.29 | 2.35 | 5.72 |
| Gm19696 | 3.44 | 6.29 | 9.47 | 16.31 |
| Gm26686 | 8.41 | 6.29 | 9.47 | 16.31 |
| Gm36161 | 2.44 | 4.11 | 1.86 | 1.58 |
| Gm36546 | 8.41 | 6.29 | 9.47 | 16.31 |
| Gm37691 | 2.84 | 6.29 | 3.78 | 1.88 |
| Gm39321 | 1.73 | 6.29 | 9.47 | 16.31 |
| Gm39459 | 2.26 | 6.29 | 9.47 | 4.22 |
| Gm40645 | 8.41 | 6.29 | 9.47 | 16.31 |
| Gm42793 | 8.41 | 6.29 | 7.48 | 5.48 |
| Gm43181 | 1.69 | 4.35 | 9.47 | 16.31 |
| Gm44165 | 3.95 | 6.29 | 9.47 | 16.31 |
| Gm45546 | 8.41 | 6.29 | 9.47 | 16.31 |
| Gm45700 | 2.09 | 6.29 | 4.93 | 16.31 |
| Gm4610 | 8.41 | 6.29 | 9.47 | 16.31 |
| Gm47069 | 8.41 | 6.29 | 9.47 | 16.31 |
| Gm4735 | 1.73 | 2.61 | 1.80 | 1.27 |
| Gm49216 | 8.41 | 6.29 | 9.47 | 16.31 |
| Gm49339 | 8.41 | 6.29 | 9.47 | 16.31 |
| Gm49759 | 1.28 | 3.23 | 1.23 | 0.72 |
| Gm5150 | 2.87 | 4.91 | 4.38 | 16.31 |
| Gm7514 | 2.61 | 3.44 | 9.47 | 16.31 |
| Gm7665 | 2.30 | 3.15 | 1.51 | 0.72 |
| Gm9888 | 8.41 | 6.29 | 9.47 | 16.31 |
| Gmfg | 1.91 | 2.78 | 2.07 | 1.94 |
| Gng8 | 8.41 | 6.29 | 2.47 | 1.55 |
| Gpr137b-ps | 2.12 | 3.03 | 0.99 | 1.06 |
| Gpr27 | 3.38 | 6.29 | 0.97 | 0.81 |
| Gpr34 | 8.41 | 6.29 | 1.73 | 1.82 |
| Gpr35 | 2.24 | 3.45 | 1.52 | 0.91 |
| Gpr84 | 8.41 | 6.29 | 9.47 | 16.31 |
| Gpr88 | 8.41 | 6.29 | 9.47 | 16.31 |
| Gpsm3 | 1.53 | 2.11 | 1.70 | 1.42 |
| Gpx7 | 8.41 | 6.29 | 1.98 | 1.67 |
| Grem1 | 8.41 | 6.29 | 1.97 | 1.68 |
| Grem2 | 8.41 | 6.29 | 1.86 | 1.62 |
| Gria3 | 8.41 | 6.29 | 1.46 | 1.38 |
| Grn | 2.76 | 3.27 | 0.75 | 0.86 |
| Gsap | 2.02 | 2.90 | 1.65 | 1.68 |
| Gsdmc4 | 8.41 | 6.29 | 3.11 | 1.72 |
| Gsdmd | 1.42 | 3.13 | 1.11 | 0.89 |
| Gsta4 | 8.41 | 6.29 | 2.59 | 1.68 |
| Gusb | 1.55 | 2.91 | 1.21 | 1.15 |
| Gxylt2 | 8.41 | 6.29 | 1.29 | 1.31 |
| Has2 | 8.41 | 6.29 | 2.82 | 2.45 |
| Has3 | 8.41 | 6.29 | 1.54 | 1.25 |
| Hbegf | 8.41 | 6.29 | 1.46 | 1.14 |
| Hcar2 | 2.22 | 3.27 | 3.01 | 2.43 |
| Hck | 2.42 | 3.82 | 2.51 | 2.21 |
| Hcls1 | 1.92 | 2.95 | 2.29 | 2.43 |
| Hdc | 2.88 | 4.11 | 3.42 | 3.02 |
| Hhex | 1.69 | 2.65 | 1.42 | 0.96 |
| Hhipl1 | 8.41 | 6.29 | 1.87 | 1.86 |
| Hic1 | 8.41 | 6.29 | 1.11 | 1.01 |
| Hif1a | 1.66 | 2.71 | 1.80 | 1.35 |
| Hk3 | 2.69 | 3.75 | 3.28 | 3.27 |
| Hmox1 | 2.14 | 3.40 | 1.03 | 1.46 |
| Hp | 3.13 | 4.06 | 1.48 | 0.75 |
| Hrh2 | 2.26 | 6.29 | 2.56 | 1.56 |
| Hsh2d | 1.61 | 6.29 | 9.47 | 3.33 |
| Ier2 | 1.56 | 2.90 | 0.78 | 0.65 |
| Ier3 | 1.68 | 2.58 | 1.94 | 1.83 |
| Ier5 | 1.71 | 2.88 | 0.59 | 0.64 |
| Ifit1bl2 | 1.94 | 2.88 | 3.04 | 3.46 |
| Ifitm1 | 4.38 | 5.39 | 2.88 | 2.68 |
| Ifitm2 | 3.07 | 4.40 | 1.16 | 1.08 |
| Ifitm6 | 5.01 | 4.83 | 2.97 | 3.07 |
| Igf1 | 8.41 | 6.29 | 1.74 | 1.35 |
| Iglon5 | 8.41 | 6.29 | 2.01 | 1.33 |
| Igsf6 | 2.39 | 3.96 | 2.85 | 2.58 |
| Ikbip | 8.41 | 6.29 | 1.50 | 1.32 |
| Il10ra | 1.23 | 2.70 | 1.55 | 1.24 |
| Il13ra1 | 2.83 | 4.45 | 1.35 | 0.89 |
| Il16 | 1.22 | 2.78 | 1.05 | 1.24 |
| Il18rap | 1.94 | 3.66 | 3.32 | 2.80 |
| Il19 | 8.41 | 6.29 | 9.47 | 2.97 |
| Il1a | 8.41 | 6.29 | 1.49 | 1.46 |
| Il1b | 2.27 | 3.96 | 5.49 | 4.69 |
| Il1r2 | 2.64 | 3.31 | 1.61 | 1.23 |
| Il1rap | 1.91 | 3.69 | 1.20 | 1.10 |
| Il33 | 8.41 | 6.29 | 3.54 | 2.94 |
| Il4ra | 1.76 | 3.29 | 2.11 | 1.56 |
| Il6 | 8.41 | 6.29 | 9.47 | 16.31 |
| Ipcef1 | 1.34 | 3.27 | 2.13 | 2.36 |
| Itga5 | 1.52 | 5.30 | 1.59 | 1.37 |
| Itgal | 1.32 | 2.58 | 1.95 | 1.94 |
| Itgam | 1.39 | 3.16 | 3.38 | 2.18 |
| Itgb2 | 2.02 | 2.63 | 2.66 | 2.25 |
| Jdp2 | 2.52 | 4.42 | 1.95 | 1.51 |
| Junb | 1.95 | 3.10 | 0.82 | 1.13 |
| Kcnc4 | 8.41 | 6.29 | 1.41 | 1.10 |
| Kcnd1 | 2.20 | 6.29 | 1.22 | 1.05 |
| Kcne4 | 8.41 | 6.29 | 1.89 | 1.79 |
| Kdelr3 | 8.41 | 6.29 | 2.37 | 2.01 |
| Klk12 | 8.41 | 6.29 | 3.04 | 2.10 |
| Klra17 | 2.85 | 4.25 | 9.47 | 16.31 |
| Klra2 | 2.24 | 3.97 | 9.47 | 16.31 |
| Krt14 | 8.41 | 6.29 | 2.84 | 1.71 |
| Krt5 | 8.41 | 6.29 | 1.62 | 1.05 |
| Krt6a | 8.41 | 6.29 | 3.37 | 1.97 |
| Lama1 | 8.41 | 6.29 | 9.47 | 1.64 |
| Lamc2 | 8.41 | 6.29 | 2.38 | 2.00 |
| Lbp | 3.16 | 3.77 | 1.25 | 1.30 |
| Lcn2 | 3.79 | 3.55 | 3.71 | 3.30 |
| Lcp1 | 1.90 | 3.07 | 2.53 | 2.56 |
| Lgi2 | 8.41 | 6.29 | 1.77 | 1.70 |
| Lhx1 | 2.46 | 6.29 | 9.47 | 16.31 |
| Lilr4b | 2.66 | 3.72 | 3.48 | 3.02 |
| Lilra6 | 2.37 | 3.70 | 9.47 | 2.77 |
| Lilrb4a | 2.68 | 4.49 | 3.60 | 3.36 |
| Lipa | 1.17 | 3.24 | 1.22 | 1.17 |
| Loxl2 | 8.41 | 6.29 | 1.88 | 1.78 |
| Lpcat2 | 2.92 | 4.47 | 0.71 | 0.85 |
| Lrch4 | 1.44 | 2.95 | 1.19 | 0.96 |
| Lrg1 | 3.28 | 4.26 | 1.51 | 1.51 |
| Lrp1 | 1.44 | 4.18 | 0.92 | 0.85 |
| Lrrc17 | 8.41 | 6.29 | 2.69 | 2.78 |
| Lrrc25 | 2.19 | 3.79 | 2.13 | 1.92 |
| Lrrk2 | 2.96 | 3.15 | 1.22 | 1.06 |
| Lsp1 | 1.83 | 3.15 | 1.45 | 1.23 |
| Lst1 | 1.40 | 2.51 | 2.78 | 2.20 |
| Ltb | 0.99 | 2.32 | 2.43 | 1.60 |
| Ltb4r1 | 2.96 | 3.45 | 1.38 | 0.64 |
| Ltf | 3.27 | 3.05 | 9.47 | 2.69 |
| Lum | 8.41 | 6.29 | 1.83 | 1.80 |
| Ly6g | 4.36 | 4.31 | 9.47 | 16.31 |
| Ly6h | 8.41 | 6.29 | 9.47 | 3.05 |
| Lyz2 | 2.64 | 2.55 | 1.82 | 1.69 |
| Mamstr | 8.41 | 6.29 | 1.41 | 1.64 |
| Marchf1 | 2.00 | 3.39 | 1.50 | 1.35 |
| Marcks | 2.05 | 3.64 | 0.90 | 0.83 |
| Marcksl1 | 2.08 | 3.02 | 0.69 | 0.78 |
| Mcam | 8.41 | 3.85 | 1.26 | 1.15 |
| Mcemp1 | 3.52 | 4.32 | 9.47 | 5.05 |
| Mctp2 | 2.19 | 4.10 | 0.78 | 0.64 |
| Mdk | 8.41 | 6.29 | 1.18 | 1.25 |
| Mefv | 2.39 | 5.10 | 5.20 | 4.18 |
| Mfap4 | 8.41 | 6.29 | 1.54 | 1.70 |
| Mgam | 3.15 | 4.29 | 4.01 | 2.05 |
| Mirg | 8.41 | 6.29 | 1.75 | 1.91 |
| Mirt1 | 1.88 | 3.69 | 2.79 | 2.73 |
| Mirt2 | 4.09 | 4.13 | 9.47 | 16.31 |
| Mlkl | 8.41 | 6.29 | 2.12 | 1.82 |
| Mmp13 | 8.41 | 6.29 | 5.40 | 3.74 |
| Mmp25 | 2.34 | 3.72 | 2.71 | 2.08 |
| Mmp8 | 3.79 | 3.93 | 9.47 | 16.31 |
| Mmp9 | 3.35 | 4.25 | 5.18 | 4.14 |
| Morc1 | 8.41 | 6.29 | 9.47 | 16.31 |
| Morc4 | 8.41 | 6.29 | 1.01 | 0.71 |
| Mov10 | 2.84 | 4.33 | 0.83 | 0.83 |
| Mpeg1 | 2.03 | 3.80 | 2.59 | 1.96 |
| Mrgpra2a | 3.22 | 6.29 | 9.47 | 16.31 |
| Mrgpra2b | 3.72 | 4.15 | 9.47 | 16.31 |
| Mrgprf | 8.41 | 6.29 | 1.53 | 1.48 |
| Ms4a4a | 2.16 | 4.07 | 1.84 | 1.28 |
| Ms4a6c | 2.11 | 3.72 | 1.69 | 1.85 |
| Ms4a6d | 2.72 | 6.29 | 2.37 | 2.11 |
| Msn | 1.21 | 2.41 | 1.24 | 1.02 |
| Msrb1 | 2.56 | 3.68 | 0.92 | 0.66 |
| Myadm | 2.08 | 3.42 | 0.81 | 0.64 |
| Mybpc3 | 2.66 | 6.29 | 3.30 | 16.31 |
| Myl4 | 8.41 | 6.29 | 1.27 | 1.16 |
| Myo1f | 2.08 | 3.65 | 1.95 | 1.73 |
| Naip2 | 2.20 | 3.90 | 2.02 | 1.80 |
| Napsa | 1.70 | 3.04 | 1.02 | 0.66 |
| Ncapg2 | 8.41 | 6.29 | 1.08 | 1.18 |
| Ncf1 | 2.79 | 3.26 | 1.45 | 1.20 |
| Ncf2 | 2.46 | 3.72 | 2.30 | 2.05 |
| Ncf4 | 2.73 | 3.14 | 2.89 | 2.30 |
| Nckap1l | 1.32 | 3.31 | 1.89 | 2.01 |
| Nedd9 | 1.77 | 3.15 | 0.67 | 1.05 |
| Neurl3 | 1.39 | 2.69 | 1.96 | 1.43 |
| Nfam1 | 1.81 | 3.49 | 1.90 | 1.95 |
| Nfe2l2 | 1.47 | 2.31 | 1.19 | 0.90 |
| Nfkbid | 1.46 | 3.03 | 3.58 | 2.76 |
| Ngp | 3.67 | 3.19 | 9.47 | 2.87 |
| Nid2 | 8.41 | 6.29 | 2.17 | 1.98 |
| Nipsnap3b | 1.98 | 2.74 | 0.89 | 0.83 |
| Nlrp12 | 2.35 | 3.90 | 3.28 | 3.06 |
| Nlrp1b | 1.98 | 3.91 | 2.11 | 0.92 |
| Nlrp3 | 1.84 | 3.31 | 4.87 | 4.03 |
| Nos2 | 8.41 | 6.29 | 5.64 | 16.31 |
| Nrg1 | 8.41 | 6.29 | 2.74 | 1.98 |
| Nrros | 2.00 | 3.21 | 1.59 | 1.39 |
| Nt5dc2 | 2.79 | 3.08 | 0.99 | 0.65 |
| Nxpe5 | 8.41 | 6.29 | 2.05 | 2.65 |
| Oaf | 8.41 | 6.29 | 1.48 | 1.46 |
| Oasl1 | 2.30 | 3.34 | 1.55 | 1.20 |
| Ocstamp | 8.41 | 6.29 | 4.37 | 16.31 |
| Olfm1 | 2.38 | 3.21 | 0.99 | 1.11 |
| Olfm4 | 3.77 | 3.44 | 4.92 | 16.31 |
| Olfml2a | 8.41 | 6.29 | 1.11 | 1.11 |
| Olfml2b | 2.98 | 2.96 | 1.44 | 1.34 |
| Osm | 1.96 | 6.29 | 5.19 | 5.76 |
| P2ry13 | 2.41 | 4.37 | 2.44 | 2.76 |
| P2ry6 | 3.05 | 6.29 | 1.54 | 1.60 |
| P3h1 | 8.41 | 6.29 | 1.77 | 1.46 |
| P3h3 | 8.41 | 6.29 | 1.42 | 1.36 |
| P4ha3 | 8.41 | 6.29 | 3.64 | 3.14 |
| Parpbp | 8.41 | 6.29 | 1.42 | 1.33 |
| Pcdh12 | 8.41 | 6.29 | 1.08 | 1.74 |
| Pcdhga11 | 8.41 | 6.29 | 9.47 | 3.77 |
| Pcdhga12 | 8.41 | 6.29 | 3.18 | 2.03 |
| Pcdhga4 | 8.41 | 6.29 | 1.95 | 2.41 |
| Pcdhga5 | 8.41 | 6.29 | 2.19 | 2.90 |
| Pcsk5 | 8.41 | 6.29 | 2.00 | 1.87 |
| Pcsk9 | 8.41 | 6.29 | 3.86 | 3.90 |
| Pde1b | 1.47 | 2.96 | 1.48 | 0.99 |
| Pdgfrb | 8.41 | 6.29 | 1.21 | 1.29 |
| Pdia5 | 8.41 | 6.29 | 1.58 | 1.36 |
| Pdpn | 8.41 | 6.29 | 1.82 | 1.80 |
| Peg12 | 8.41 | 6.29 | 2.28 | 1.45 |
| Pfn1 | 1.50 | 2.16 | 0.79 | 0.94 |
| Pgam1 | 1.91 | 2.78 | 1.02 | 0.78 |
| Pgf | 8.41 | 6.29 | 2.13 | 1.72 |
| Pglyrp1 | 3.50 | 3.91 | 2.47 | 2.36 |
| Pi15 | 8.41 | 6.29 | 1.22 | 1.16 |
| Pik3ap1 | 1.30 | 3.23 | 2.91 | 2.92 |
| Pik3cd | 1.01 | 2.58 | 1.29 | 1.45 |
| Pik3cg | 1.75 | 3.27 | 1.31 | 1.00 |
| Pik3r5 | 1.27 | 2.83 | 1.85 | 1.30 |
| Pilra | 2.77 | 3.84 | 3.94 | 3.62 |
| Pilrb1 | 2.33 | 3.14 | 9.47 | 1.78 |
| Pilrb2 | 2.41 | 4.10 | 3.70 | 2.56 |
| Pip5k1c | 1.62 | 3.03 | 0.83 | 1.06 |
| Pirb | 2.76 | 4.18 | 2.39 | 2.09 |
| Pla2g15 | 2.31 | 3.75 | 0.83 | 1.11 |
| Pla2g7 | 2.26 | 3.89 | 2.00 | 1.89 |
| Plagl1 | 8.41 | 6.29 | 1.14 | 1.11 |
| Plaur | 2.89 | 3.93 | 4.18 | 3.44 |
| Pld1 | 3.04 | 3.48 | 1.22 | 0.74 |
| Plekhg4 | 8.41 | 6.29 | 9.47 | 2.17 |
| Plxna4os1 | 8.41 | 6.29 | 9.47 | 16.31 |
| Ppp1r3d | 3.12 | 4.06 | 0.73 | 1.03 |
| Pram1 | 3.88 | 4.38 | 2.12 | 1.65 |
| Prex1 | 1.25 | 2.38 | 1.29 | 1.06 |
| Prok2 | 8.41 | 6.29 | 9.47 | 16.31 |
| Prokr2 | 8.41 | 6.29 | 3.15 | 2.43 |
| Prss35 | 8.41 | 6.29 | 2.62 | 2.14 |
| Pstpip1 | 1.84 | 3.16 | 0.92 | 1.14 |
| Ptafr | 1.89 | 3.81 | 2.10 | 2.30 |
| Pth1r | 8.41 | 6.29 | 1.04 | 1.04 |
| Ptpn6 | 1.64 | 3.13 | 1.11 | 0.97 |
| Ptprc | 1.02 | 2.64 | 2.49 | 2.16 |
| Ptprz1 | 8.41 | 6.29 | 3.35 | 2.12 |
| Rab20 | 1.83 | 5.00 | 2.43 | 2.33 |
| Rab31 | 2.70 | 4.02 | 1.00 | 1.10 |
| Rab44 | 2.09 | 3.10 | 1.10 | 1.30 |
| Rac2 | 2.06 | 3.12 | 2.54 | 2.28 |
| Raet1d | 8.41 | 6.29 | 2.87 | 2.80 |
| Raet1e | 8.41 | 6.29 | 9.47 | 16.31 |
| Ralb | 1.66 | 2.47 | 1.01 | 1.03 |
| Rbp1 | 8.41 | 6.29 | 1.67 | 1.41 |
| Relt | 1.85 | 3.41 | 1.36 | 1.54 |
| Rem1 | 8.41 | 6.29 | 1.31 | 1.49 |
| Retnlg | 3.79 | 4.46 | 6.39 | 4.96 |
| Rgs14 | 1.44 | 2.75 | 0.61 | 0.74 |
| Rgs16 | 8.41 | 6.29 | 1.12 | 1.15 |
| Rgs19 | 1.93 | 2.81 | 1.11 | 1.00 |
| Rnd1 | 3.16 | 4.34 | 1.18 | 0.87 |
| Rnf144a | 2.21 | 3.97 | 0.62 | 0.67 |
| Rnpep | 2.35 | 3.22 | 0.66 | 0.61 |
| Rpl39l | 8.41 | 6.29 | 1.60 | 1.25 |
| Rrbp1 | 1.14 | 2.49 | 0.83 | 0.85 |
| Rtl1 | 8.41 | 6.29 | 2.96 | 2.73 |
| Rubcnl | 1.73 | 6.29 | 1.50 | 1.27 |
| S100a11 | 2.84 | 3.93 | 1.30 | 0.66 |
| S100a8 | 3.96 | 4.31 | 6.03 | 4.64 |
| S100a9 | 3.64 | 4.22 | 6.36 | 5.17 |
| Saa3 | 8.41 | 6.39 | 5.85 | 4.65 |
| Samd12 | 8.41 | 6.29 | 2.06 | 1.35 |
| Samd9l | 1.10 | 2.77 | 1.18 | 1.00 |
| Samhd1 | 1.42 | 2.88 | 1.50 | 0.83 |
| Samsn1 | 1.92 | 3.24 | 5.07 | 4.16 |
| Sash3 | 1.14 | 2.65 | 1.75 | 1.45 |
| Sbno2 | 2.39 | 3.82 | 1.17 | 0.85 |
| Scarf2 | 8.41 | 6.29 | 1.87 | 1.73 |
| Sdk1 | 8.41 | 6.29 | 1.20 | 1.02 |
| Sec16b | 8.41 | 6.29 | 1.71 | 1.48 |
| Sele | 8.41 | 6.29 | 2.81 | 2.20 |
| Selenon | 2.75 | 3.41 | 1.27 | 1.23 |
| Sell | 1.34 | 2.92 | 4.41 | 4.39 |
| Selplg | 2.17 | 3.09 | 1.87 | 1.60 |
| Sema3a | 8.41 | 6.29 | 2.04 | 1.51 |
| Sema4d | 1.05 | 2.52 | 0.61 | 0.64 |
| Sema6b | 3.21 | 4.35 | 1.37 | 0.91 |
| Serpina3n | 8.41 | 6.29 | 2.48 | 1.65 |
| Serpina9 | 8.41 | 6.29 | 2.84 | 2.85 |
| Serpinb10 | 2.19 | 3.39 | 1.43 | 0.91 |
| Serpinb1a | 2.04 | 2.86 | 1.52 | 1.25 |
| Sfxn5 | 2.42 | 4.30 | 0.75 | 0.68 |
| Sgms2 | 2.51 | 5.21 | 1.15 | 1.30 |
| Sh2d3c | 1.76 | 3.30 | 2.56 | 2.53 |
| Sh3bp2 | 1.94 | 3.48 | 1.46 | 1.17 |
| Siglece | 2.82 | 3.92 | 3.37 | 2.70 |
| Sirpa | 1.86 | 2.80 | 1.05 | 1.13 |
| Sirpb1a | 2.90 | 4.27 | 9.47 | 16.31 |
| Sirpb1b | 8.41 | 6.29 | 9.47 | 16.31 |
| Sirpb1c | 3.12 | 4.48 | 9.47 | 5.24 |
| Slc11a1 | 2.03 | 3.76 | 3.04 | 2.69 |
| Slc15a3 | 2.46 | 3.58 | 4.06 | 3.27 |
| Slc16a3 | 3.13 | 4.24 | 1.77 | 2.04 |
| Slc28a2 | 2.42 | 4.14 | 1.71 | 1.89 |
| Slc28a2b | 2.02 | 3.19 | 1.50 | 1.56 |
| Slc2a1 | 2.23 | 3.65 | 2.76 | 1.92 |
| Slc2a10 | 8.41 | 6.29 | 1.69 | 1.23 |
| Slc2a6 | 2.74 | 4.06 | 2.91 | 3.02 |
| Slc32a1 | 8.41 | 6.29 | 9.47 | 16.31 |
| Slc7a7 | 2.54 | 3.17 | 1.28 | 0.93 |
| Slc9b2 | 8.41 | 6.29 | 3.01 | 2.01 |
| Slfn1 | 1.95 | 3.65 | 9.47 | 3.42 |
| Slfn10-ps | 8.41 | 6.29 | 1.88 | 1.82 |
| Slfn2 | 1.65 | 2.97 | 2.94 | 2.45 |
| Slfn4 | 3.02 | 4.31 | 4.48 | 3.62 |
| Slpi | 2.79 | 3.47 | 4.13 | 2.71 |
| Smpd5 | 3.22 | 4.34 | 2.19 | 2.75 |
| Snai1 | 8.41 | 6.29 | 2.03 | 1.86 |
| Snx20 | 1.63 | 3.04 | 2.29 | 2.01 |
| Soat2 | 3.03 | 6.29 | 2.97 | 3.41 |
| Socs3 | 2.41 | 3.69 | 2.54 | 1.92 |
| Sorl1 | 1.75 | 3.51 | 0.67 | 0.75 |
| Sox11 | 8.41 | 6.29 | 2.35 | 1.17 |
| Sp140 | 2.03 | 3.47 | 2.59 | 1.78 |
| Spats2l | 8.41 | 6.29 | 1.76 | 1.77 |
| Spi1 | 2.68 | 3.89 | 2.53 | 1.99 |
| Spic | 8.41 | 6.29 | 2.45 | 2.09 |
| Spon1 | 8.41 | 6.29 | 1.71 | 1.84 |
| St8sia2 | 8.41 | 6.29 | 1.57 | 1.36 |
| St8sia4 | 1.43 | 3.13 | 1.33 | 1.40 |
| Stat3 | 1.49 | 3.00 | 1.01 | 0.59 |
| Steap4 | 3.23 | 4.91 | 1.31 | 1.20 |
| Stfa1 | 8.41 | 6.29 | 4.51 | 1.78 |
| Stfa2 | 5.48 | 5.06 | 4.82 | 2.28 |
| Stfa2l1 | 3.27 | 4.26 | 9.47 | 16.31 |
| Stfa3 | 8.41 | 6.29 | 3.66 | 1.61 |
| Sult6b2 | 8.41 | 6.29 | 9.47 | 16.31 |
| Sv2c | 8.41 | 6.29 | 2.84 | 2.10 |
| Svep1 | 8.41 | 6.29 | 1.65 | 1.48 |
| Swap70 | 1.30 | 3.09 | 0.60 | 0.69 |
| Sycp2 | 2.22 | 4.49 | 9.47 | 2.14 |
| Syk | 1.59 | 2.45 | 0.90 | 0.92 |
| Tarm1 | 3.77 | 6.29 | 3.98 | 3.28 |
| Tbc1d2 | 3.25 | 3.74 | 1.10 | 0.84 |
| Tbc1d2b | 1.62 | 2.51 | 0.98 | 1.03 |
| Teddm3 | 8.41 | 6.29 | 2.51 | 1.33 |
| Tenm3 | 8.41 | 6.29 | 1.49 | 1.49 |
| Tfpi2 | 8.41 | 6.29 | 3.50 | 2.53 |
| Tgfbi | 2.78 | 4.28 | 1.14 | 0.97 |
| Thbs2 | 8.41 | 6.29 | 1.72 | 1.71 |
| Thbs4 | 8.41 | 6.29 | 1.74 | 2.65 |
| Themis2 | 1.97 | 3.69 | 2.40 | 2.60 |
| Tie1 | 8.41 | 6.29 | 1.52 | 1.06 |
| Tifab | 2.06 | 3.80 | 2.12 | 1.99 |
| Timp1 | 8.41 | 6.29 | 4.75 | 3.55 |
| Tiparp | 1.36 | 2.73 | 1.22 | 1.04 |
| Tlr1 | 2.13 | 3.69 | 3.96 | 4.00 |
| Tlr13 | 2.49 | 3.86 | 3.21 | 3.14 |
| Tlr2 | 3.24 | 4.48 | 2.99 | 2.66 |
| Tlr4 | 1.62 | 3.77 | 1.00 | 0.93 |
| Tlr6 | 2.05 | 3.32 | 2.65 | 2.38 |
| Tlr8 | 2.50 | 4.00 | 1.85 | 1.23 |
| Tmem119 | 8.41 | 6.29 | 1.78 | 1.34 |
| Tmem26 | 8.41 | 6.29 | 2.01 | 2.12 |
| Tmem88 | 8.41 | 6.29 | 1.78 | 1.54 |
| Tmprss11a | 8.41 | 6.29 | 2.92 | 1.86 |
| Tmprss11d | 8.41 | 6.29 | 4.71 | 2.87 |
| Tmprss11g | 8.41 | 6.29 | 2.74 | 1.69 |
| Tnc | 8.41 | 6.29 | 4.11 | 3.61 |
| Tnfaip2 | 2.68 | 4.41 | 1.74 | 2.00 |
| Tnfaip6 | 8.41 | 6.29 | 1.62 | 1.46 |
| Tnfaip8l2 | 2.04 | 2.90 | 1.21 | 1.36 |
| Tnfrsf1b | 1.62 | 3.36 | 1.82 | 1.67 |
| Tnfrsf23 | 1.95 | 6.29 | 2.30 | 1.84 |
| Tnfsf11 | 8.41 | 6.29 | 9.47 | 16.31 |
| Tnfsf14 | 2.28 | 3.63 | 3.19 | 2.71 |
| Tnn | 8.41 | 6.29 | 6.16 | 4.28 |
| Tor4a | 1.41 | 2.81 | 0.80 | 0.60 |
| Tpcn2 | 1.98 | 3.21 | 1.07 | 0.78 |
| Tpd52 | 2.33 | 3.17 | 0.63 | 0.87 |
| Trem1 | 2.36 | 3.16 | 9.47 | 4.98 |
| Trem3 | 3.57 | 3.94 | 9.47 | 16.31 |
| Treml4 | 1.57 | 3.28 | 3.54 | 16.31 |
| Trim25 | 1.30 | 3.16 | 1.75 | 1.56 |
| Trim30a | 1.77 | 3.31 | 1.43 | 1.32 |
| Trim30b | 2.10 | 4.36 | 3.88 | 2.91 |
| Tro | 8.41 | 6.29 | 1.93 | 1.67 |
| Trpm2 | 2.04 | 3.60 | 4.07 | 2.54 |
| Tslp | 8.41 | 6.29 | 1.29 | 1.89 |
| Tspan11 | 8.41 | 6.29 | 1.31 | 1.19 |
| Txn1 | 2.65 | 3.35 | 0.90 | 0.70 |
| Tyrobp | 2.53 | 3.79 | 2.23 | 2.30 |
| Ucn2 | 8.41 | 6.29 | 2.00 | 1.06 |
| Unc5c | 8.41 | 6.29 | 3.23 | 1.88 |
| Unc93b1 | 1.79 | 3.32 | 1.39 | 1.17 |
| Upp1 | 4.55 | 4.92 | 1.41 | 2.51 |
| Vash2 | 8.41 | 6.29 | 1.49 | 1.48 |
| Vasp | 1.52 | 2.41 | 1.16 | 1.03 |
| Vav1 | 1.64 | 2.94 | 2.19 | 1.87 |
| Vcan | 3.39 | 6.29 | 1.02 | 1.05 |
| Vsnl1 | 8.41 | 6.29 | 1.63 | 1.51 |
| Vstm4 | 8.41 | 6.29 | 1.60 | 1.37 |
| Was | 1.34 | 2.35 | 2.01 | 0.96 |
| Wdfy4 | 1.53 | 3.18 | 1.68 | 1.55 |
| Wfdc17 | 5.33 | 7.48 | 3.03 | 2.45 |
| Wsb1 | 1.71 | 3.20 | 0.99 | 0.67 |
| Xylt1 | 1.74 | 3.55 | 1.04 | 1.01 |
| Zc3h12d | 1.61 | 3.24 | 1.46 | 1.44 |
| Zfp36 | 1.78 | 2.99 | 0.94 | 0.78 |
| Zfp469 | 8.41 | 6.29 | 2.39 | 2.04 |
