# Supplementary figures and images for: Human umbilical cord mesenchymal stem cell treatment alleviates symptoms in an atopic dermatitis-like mouse model
Source: Stem Cell Res Ther. 2023 May 29;14:147. doi: 10.1186/s13287-023-03365-w (PMC10227992; doi:10.1186/s13287-023-03365-w)

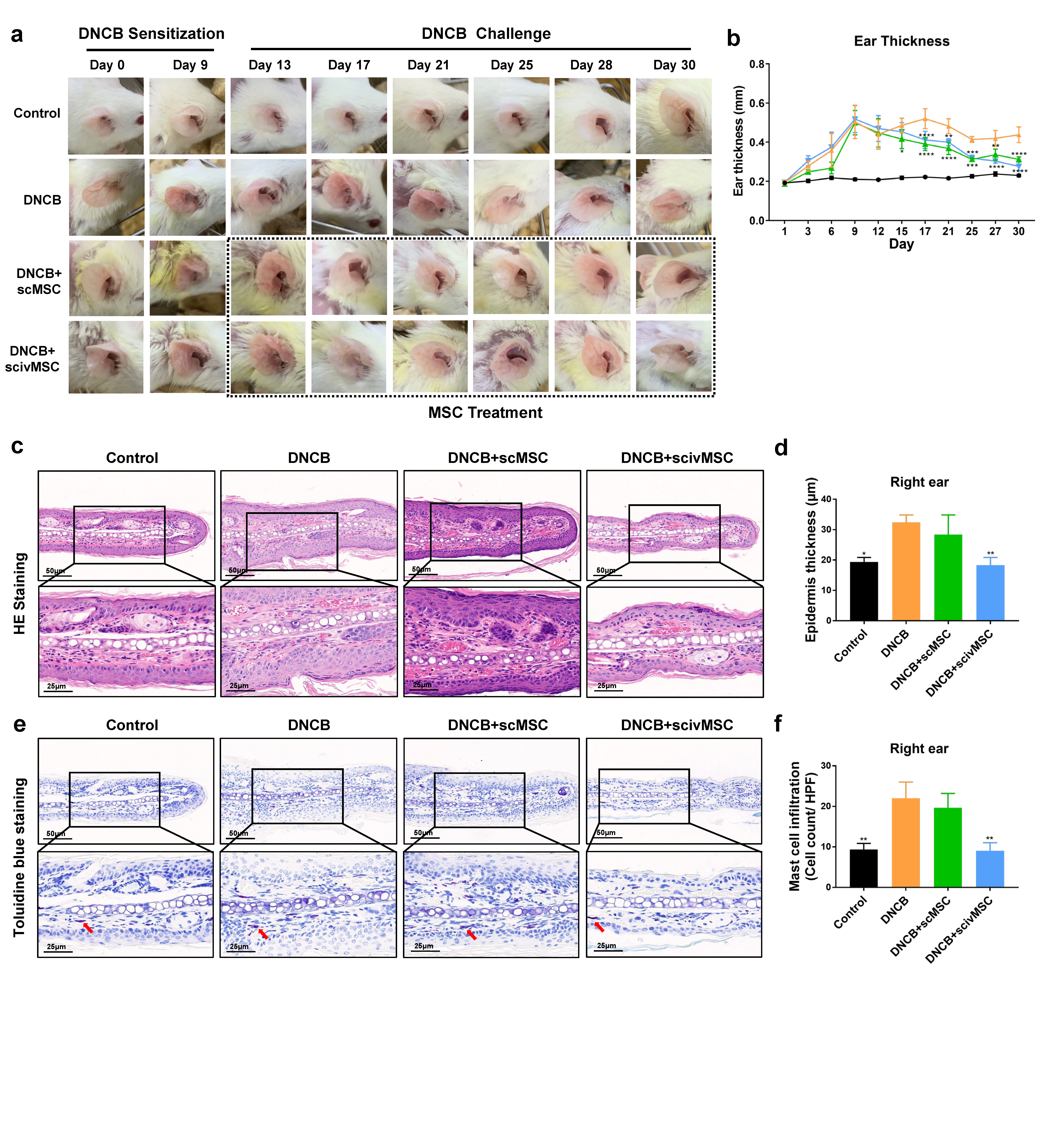

Supplement: Supplementary file 2 — Additional file 2: Fig. S1. Morphology and pathological changes of right ear and its mast cell infiltration. a Macroscopic views of right ear appearance from representative mice in each group from day 0 to day 30. b Record of ear thickness during DNCB application and MSC treatment. c HE staining of right ear in different groups. d Measurements of epidermis thickness of right ear based on slides as presented in panel c. e Toluidine blue staining of right ear in different groups. f Mast cells infiltration of right ear based on slides as presented in panel. One-way ANOVA with Tukey’s multiple comparison test was used to test statistical significance. *, P < 0.05; **, P < 0.01; ***, P < 0.001; ****, P < 0.0001. [file 13287_2023_3365_MOESM2_ESM.tiff]

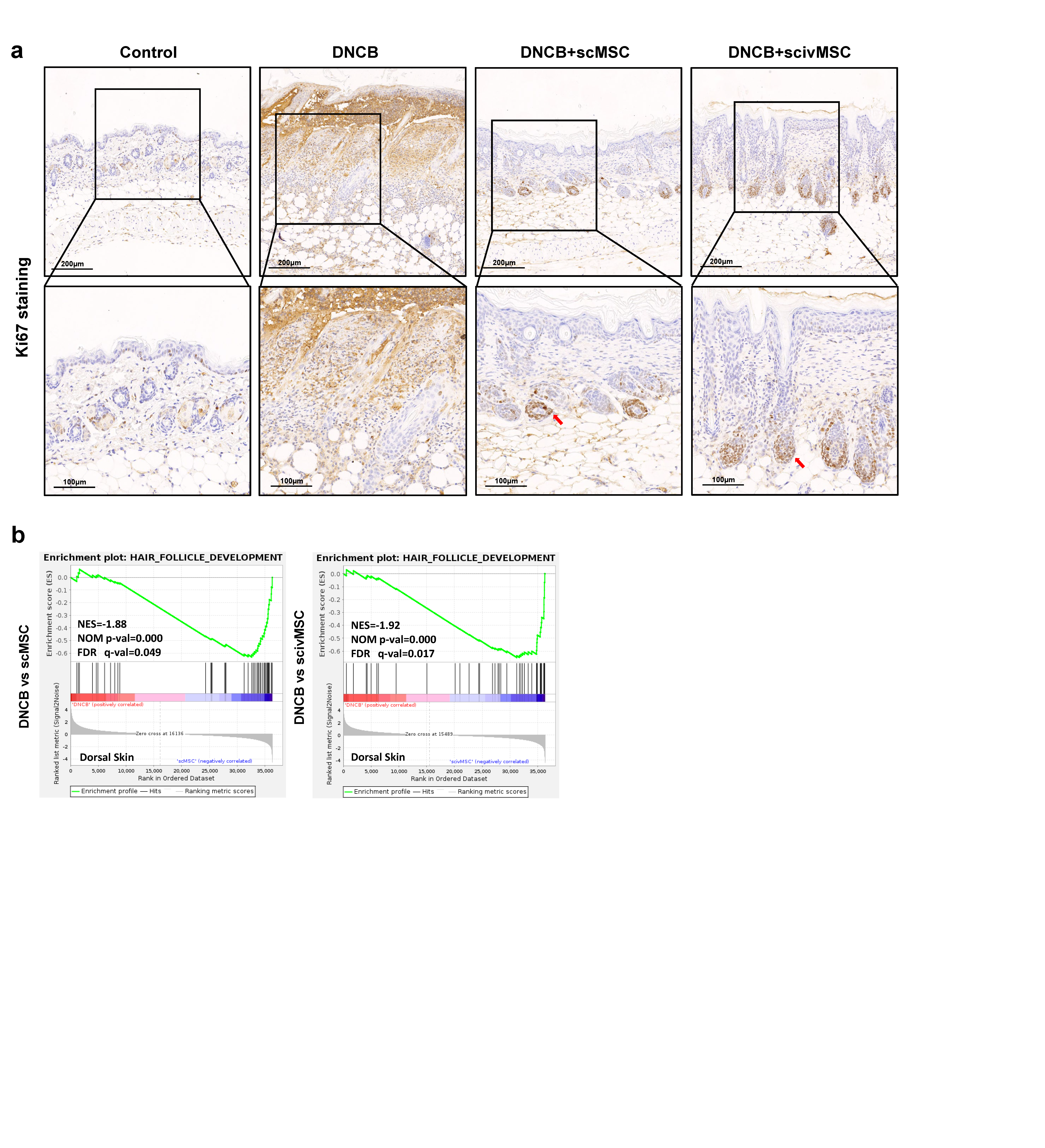

Supplement: Supplementary file 3 — Additional file 3: Fig. S2. Effects on hair growth by MSC treatment in DNCB mice model. a Ki67 staining of dorsal skin sections. Positive staining indicates activated proliferation. b GSEA analysis related to hair follicle development of dorsal skin between DNCB and MSC treatment groups. One-way ANOVA with Tukey’s multiple comparison test was used to test statistical significance. *, P < 0.05; **, P < 0.01; ***, P < 0.001; ****, P < 0.0001. [file 13287_2023_3365_MOESM3_ESM.tiff]

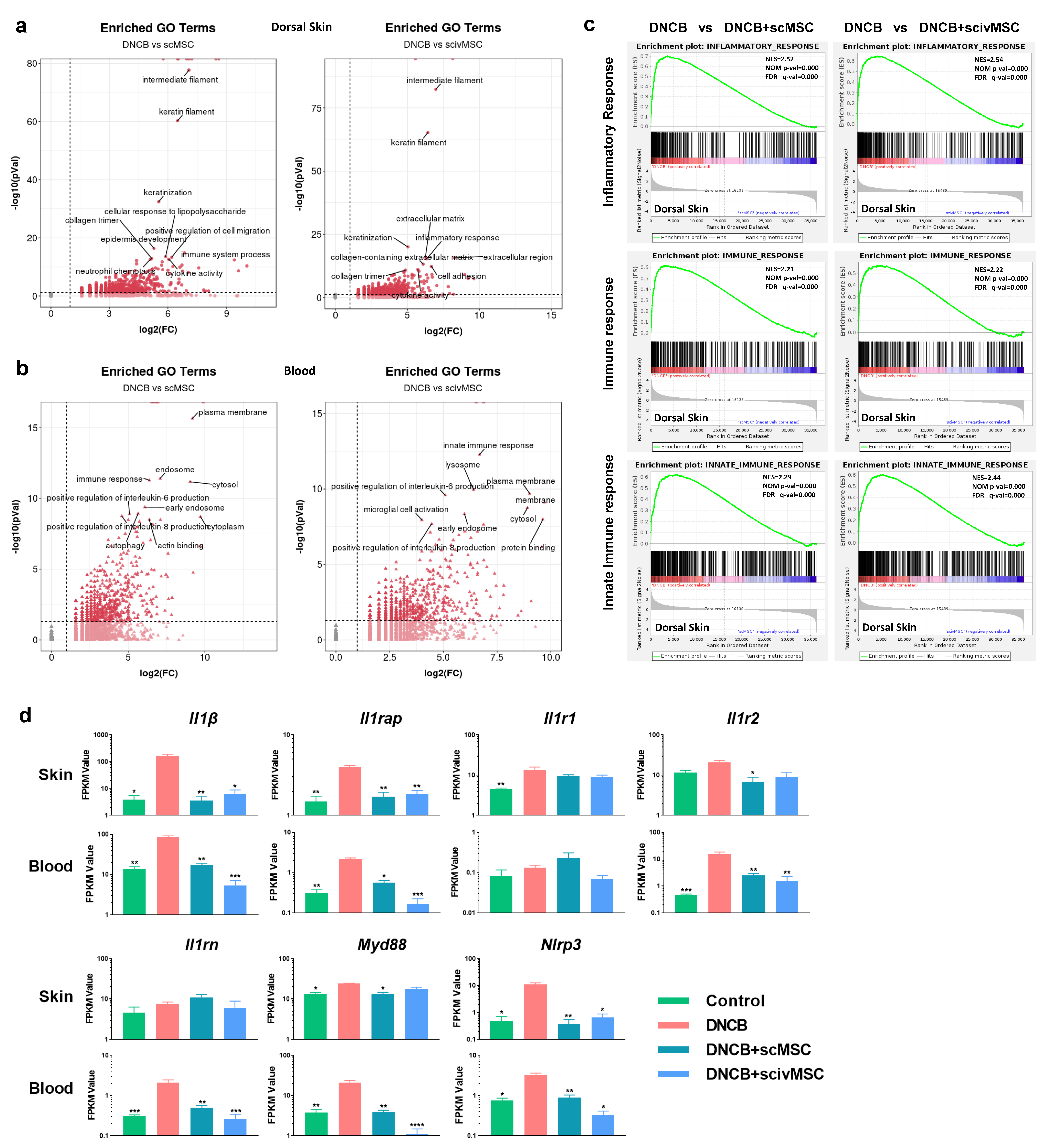

Supplement: Supplementary file 4 — Additional file 4: Fig. S3. Inflammatory and immune patterns changes based on RNA Seq. a GO enrichment analysis of dorsal skin between DNCB vs scMSC and DNCB vs scivMSC. b GO enrichment analysis of blood between DNCB vs scMSC and DNCB vs scivMSC. c GSEA analysis related to inflammatory, immune and innate immune response of dorsal skin between DNCB vs scMSC and DNCB vs scivMSC. d Transcriptome changes of IL1β, IL1RAP, IL1R1, IL1R2, IL1RN, Myd88 and Nlrp3 in dorsal skin and blood. One-way ANOVA with Tukey’s multiple comparison test was used to test statistical significance. *, P < 0.05; **, P < 0.01; ***, P < 0.001; ****, P < 0.0001. [file 13287_2023_3365_MOESM4_ESM.tiff]

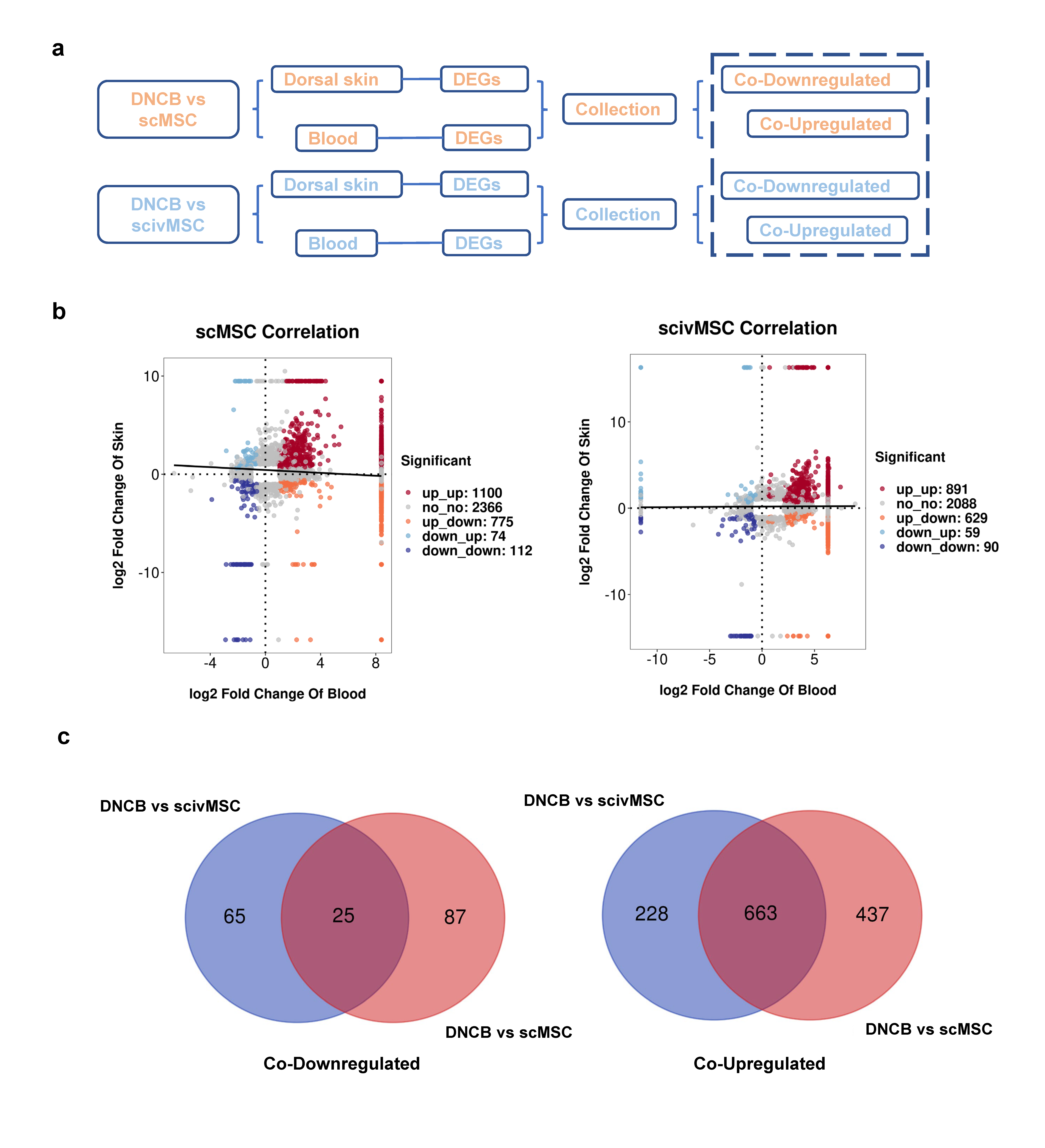

Supplement: Supplementary file 5 — Additional file 5: Fig. S4. Process of picking common AD signatures. a Secondary analysis of differentially expressed genes between DNCB vs scMSC and DNCB vs scivMSC in dorsal skin and blood. b Advanced volcano plots show gene correlation with different MSC treatment routes.Collection of genes with same variation tendency in different MSC treatment routes. [file 13287_2023_3365_MOESM5_ESM.tiff]
